# Dynamic formation of cellular aggregates of chondrocytes and mesenchymal stem cells in spinner flask

**DOI:** 10.1111/cpr.12587

**Published:** 2019-06-17

**Authors:** Huimin He, Qing He, Feiyue Xu, Yan Zhou, Zhaoyang Ye, Wen‐Song Tan

**Affiliations:** ^1^ The State Kay Laboratory of Bioreactor Engineering East China University of Science and Technology Shanghai China

**Keywords:** aggregation kinetics, articular chondrocytes, cell aggregation, mesenchymal stem cells, spinner flask

## Abstract

**Objectives:**

Cellular aggregates are readily applicable in cell‐based therapy. The effects of agitation and inoculation density on the aggregation of cells in spinner flask and the molecular mechanism of aggregation were investigated.

**Materials and methods:**

The aggregation kinetics of cells in spinner flask was evaluated with bovine articular chondrocytes (bACs), rabbit bone marrow‐derived mesenchymal stem cells (rMSCs) and their mixture. The morphology of cellular aggregates was studied with scanning electron microscopy and gene expression of cell adhesion‐related molecules was analysed.

**Results:**

It was shown that suspension culture in spinner flask induced the aggregation of bACs and rMSCs. Both cells exhibited increased aggregation rate and aggregate size with decreasing agitation rate and increasing cell inoculation density. Additionally, aggregate size increased with extended culture time. By analysing gene expression of integrin β1 and cadherin, it was indicated that these molecules were potentially involved in the aggregation process of bACs and rMSCs, respectively. Aggregates composed of both bACs and rMSCs were also prepared, showing rMSCs in the core and bACs in the periphery.

**Conclusions:**

Cellular aggregates were prepared in dynamic suspension culture using spinner flask, the key parameters to the aggregation process were identified, and the molecular mechanism of aggregation was revealed. This would lay a solid foundation for the large‐scale production of cellular aggregates for cell‐based therapy, such as cartilage regeneration.

## 1 INTRODUCTION

Cell‐based therapies using culture‐expanded chondrocytes and mesenchymal stem cells (MSCs) are emerging as a promising route for cartilage regeneration.^1^, ^2^ However, expansion of chondrocytes in two‐dimensional (2D) substratum generally leads to dedifferentiation, characterized by decreased proteoglycan synthesis and hyaline cartilage‐specific type II collagen expression and increased fibrotic type Ι collagen expression.^3^ Moreover, MSCs gradually eventually lose their differentiation potential, colony‐forming efficiency and self‐renewal capacity upon successive passages in 2D culture.^4^ New strategies for cell expansion are urgently needed.

Cell culture in three‐dimensional (3D) fashion is receiving increased interest, with evidence showing that 3D cell culture creates an artificial microenvironment that mimics the in vivo biological cues.^5^ There are many approaches to achieve 3D cell culture, including use of hydrogels and 3D scaffolds and formation of multicellular aggregates.^4^ For instance, autologous nasal chondrocytes suspended in alginate hydrogel as injectable constructs for rabbit articular cartilage repair obtained superior and more hyaline‐like repaired tissue, demonstrating similar mechanical properties to native cartilage^6^; dedifferentiated chondrocytes regained a functional chondrocyte phenotype when embedded in a 3D porous scaffold made of alginate and gelatin^7^; when transplantation of aggregates of synovial MSCs formed by hanging drop regenerated meniscus more effectively than intra‐articular injection of suspension of synovial MSCs with the same number of cells in a rat massive meniscal defect.^8^

Among the above approaches, cellular aggregates not only provide an in vitro culture condition mimicking in vivo microenvironment, which allow cell‐cell contact and cell‐ECM interactions, but also are readily applicable in cell‐based therapy.^9^ The exquisite communication network of mechanical and biochemical signals within 3D cellular aggregates is critical for improving cellular viability, phenotype and function that are often lost in 2D culture.^10^ As early as the 1960s, it was shown that monodispersed chondrocytes spontaneously formed aggregates in suspension culture and secreted abundant collagen II and glycosaminoglycans (GAG), typical matrix components in hyaline cartilage.^11^ Recently, Bhumiratana et al^12^ proposed that small spheroids of MSCs could be induced to fuse and form mechanically functional human cartilage in vitro by mimicking some aspects of the pivotal stage during chondrogenesis, which has potential use for repairing cartilage defects.

Approaches to fabricate cellular aggregates including liquid overlap technique, handing drop and microfluidics as well as bioreactors have been reported.^13^ Among these, the advantages of bioreactors lie in conveniently generating a large quantity of cellular aggregates, providing a controlled dynamic fluid environment that will increase the mass transfer of nutrients, oxygen and metabolites and producing the mechanical stimulation such as shear stress on cells, which can potentially influence cell phenotype.^14^ A series of reports have indicated the formation of chondrocytes and MSCs aggregates in suspension culture in bioreactors. Lee et al^15^ showed that spinner flask culture induced formation of articular chondrocyte aggregates and, consequently, re‐differentiation of the dedifferentiated chondrocytes. Frith et al^4^ showed that in vitro culture of MSCs in spinner flask induced the formation of compact cellular spheroids, displaying great cell viability and multipotent differentiation potential.

In the present study, culture of bovine articular chondrocytes (bACs), rabbit mesenchymal stem cells (rMSCs) and mixed cell population of bACs and rMSCs in spider flask was established and the effect of agitation rate and cell inoculation density on the kinetics of aggregation as well as the associated molecular mechanism of aggregation were explored. The key parameters to the aggregation process were identified, and the molecular mechanism of aggregation was revealed. This would lay a solid foundation for large‐scale production of cellular aggregates for cell‐based therapy, such as cartilage regeneration.

## 2 MATERIALS AND METHODS

### 2.1 Cell isolation and monolayer culture

Under aseptic conditions, full‐thickness articular cartilage tissue was obtained from bovine tibiae and femora. The tissue was minced into small slices, washed three times in phosphate‐buffered saline (PBS), and digested with 0.25% trypsin (Invitrogen, Carlsbad, CA, USA) for 20 minutes and 0.2% (w/v) collagenase type II (Invitrogen) in Dulbecco’s modified Eagle medium containing (DMEM; Gibco, Grand Island, NY, USA) 10% (v/v) fetal bovine serum (FBS; Biosun, Shanghai, China) for 6‐8 hours in cell culture incubator. The resulting chondrocyte suspension was filtered with a 100‐mesh sieve to remove the undigested tissue. Single cells were inoculated on culture flasks with chondrocyte culture medium consisted of DMEM supplemented with 10% FBS, 0.1 mmol/L non‐essential amino acid (Sigma, St. Louis, MO, USA), 40 μg/mL l‐Pro (Sigma), 50 μg/mL l‐ascorbic acid (Sigma), 100 U/mL penicillin and 100 μg/mL streptomycin in a 37°C, 5% CO_2 _incubator. The medium was changed after 3 days and twice per week thereafter until the cells reached 80%‐90% confluence. Cells were detached using 0.25% (w/v) trypsin/PBS containing 0.02% (w/v) ethyl enediamine tetra acetic acid (EDTA; Gibco), resuspended with fresh medium after centrifuging at 371 *g* for 5 minutes, and replated at 5 × 10^3 ^cells/cm^2^. Passage 2 (P2) cells were used for culture in spinner flask.

Rabbit tibiae and femura were obtained from 4‐ to 6‐week‐old New Zealand white rabbits. Bone marrow was flushed into sterile Petri dishes with PBS, transferred into a conical tube, and mixed with an equal volume of Ficoll‐Paque (GE, Little Chalfont, Buckinghamshire, UK). The mixture was centrifuged at 1284 *g* for 10 minutes. Mononuclear cells were harvested and seeded into culture flasks with Minimum Essential Medium Alpha Medium (α‐MEM; Gibco) supplemented with 10% FBS, 100 IU/mL penicillin and 100 μg/mL streptomycin. Non‐adherent cells were removed after 72 hours. When reaching 80%‐90% confluence, MSCs were detached and replated. Passage 5 (P5) cells were used for subsequent culture in spinner flask.

All animal experiments were performed in accordance with the guidelines for the care and use of laboratory animals at Shanghai Laboratory Animal Center (Shanghai), and the protocols were approved by the institutional animal care and use committee of Shanghai Laboratory Animal Center.

### 2.2 Cell labelling

To distinguish the different cells in the mixture of bACs and rMSCs, the harvested P2 bACs and P5 rMSCs were labelled with CFSE (Sigma) and PKH26 (Dojindo, Kumamoto, Japan) according to the manufacturer’s instructions, respectively. CFSE was capable of penetrating into cell membrane, covalently binding to intracellular proteins, and releasing green fluorescence after hydrolyzation. PKH26 was stably incorporated and retained in plasma membrane, red fluorescence.

### 2.3 Cell culture in spinner flask

Spinner flasks (Corning) were silicon‐treated to prevent cells from adhering to the flask walls. Cells were seeded into spinner flasks while keeping total volume at 100 mL and cultured for 5 days. The medium was kept during the process of any operations on cellular aggregates, and the sidearm caps of spinner flasks were loosened to allow for gas exchange.

P2 bACs of 4 × 10^5^ cells/mL were seeded into spinner flasks at three different agitation rates (40, 50 and 60 rpm) to investigate the effect of agitation rate on the formation of cellular aggregates. And cells were also seeded into spinner flasks in three different seeding densities (2, 4 and 8 × 10^5^ cells/mL) while keeping the agitation rate of 50 rpm.

P5 rMSCs were seeded at the same densities as bACs at an agitation rate of 45 rpm to investigate the effect of cell seeding density on cell aggregates, while the three agitations rates were separately 40, 45 and 50 rpm to evaluate the effect of agitation rate.

Additionally, labelled P2 bACs and P5 rMSCs were mixed at a ratio of 1:1 and seeded into spinner flask at an agitation rate of 40 rpm with a total cell density of 2 × 10^5^ cells/mL.

### 2.4 Cell counting and image analyses

After 0, 2, 4, 6, 8, 10, 12, 22 and 24 hours of culture in spinner flask, 300 μL aliquots of cell suspension in triplicate were harvested. The samples were used to count single cells at each time point (*C*_t_) with a hemocytometer. The proportion of aggregated cells at each time point was calculated using the following formula (1). It was assumed that total cell concentration (*C*_0_), which equalled to the cell seeding density, remained constant, which was verified by determining DNA content and MTT assay between 0 and 1 day (data not shown). Based on the loss of single cells from the suspension with time, the aggregation kinetics was calculated using the following formula (1), which is based on the mathematical model proposed by Morris.^16^

(1) $$\%\;\text{Aggregates}=\frac{C_{0}-C_{t}}{C_{0}}\times 100\%$$

(2) $$\frac{-\partial C_{t}}{\partial t}=k\times C_{t}$$

(3) $$\ln\frac{C_{t}}{C_{0}}=-kt$$

where *k* [h^−1^] is the kinetic constant of cell incorporation into cellular aggregates.

In addition, 200 μL aliquots of cell suspension in triplicate were obtained after 1, 3 and 5 days of culture and imaged under inverted microscope (Eclipse Ti, Nikon, Japan). Then, the size distribution and roundness distribution of these images were analysed by Image J software (n = 500‐1000 per time point).

### 2.5 Scanning electron microscopy

For scanning electron microscopy (SEM), samples taken after 1, 3 and 5 days of culture in spinner flask were washed twice with PBS, fixed in 2.5% glutaraldehyde at 4°C overnight, dehydrated in ascending grades of ethanol and air dried. The samples were fixed, sputter‐coated with gold and examined under SEM (S‐3400; Hi‐tachi, Tokyo, Japan).

### 2.6 Confocal laser scanning microscopy

To evaluate the location and distribution of cells in the aggregate composed of bACs and rMSCs, the aggregates after 1, 3 and 5 days of culture in spinner flask were observed using confocal laser scanning microscopy (CLSM; TI‐LU4SU, Nikon, Japan).

### 2.7 Quantitative real‐time reverse transcription‐polymerase chain reaction (qRT‐PCR)

To examine gene expression of cells, total RNA was extracted by Trizol (Invitrogen) reagent. The concentration of recovered RNA was determined by Nanodrop (ND‐2000; Thermo, Waltham, MA, USA). Following the manufacturer’s protocol, cDNAs were synthesized from 1 µg of total RNA. Real‐time PCR was performed using SYBR Green qPCR kit (Takara, Tokyo, Japan) on a thermal cycler (Mx3000P; Biorad, Hercules, CA, USA). The primer sequences for bovine and rabbit gene used were listed in Tables S1 and S2, respectively. The expression levels of mRNA were calculated and normalized to GAPDH.

### 2.8 Statistical analysis

All quantitative results were represented as mean ± standard deviation. Student’s *t *test was used to analyse the significant difference between two groups. Significance was defined as *P* < 0.05.

## 3 RESULTS

### 3.1 Formation of bACs aggregates in spinner flask

**Figure 1:**
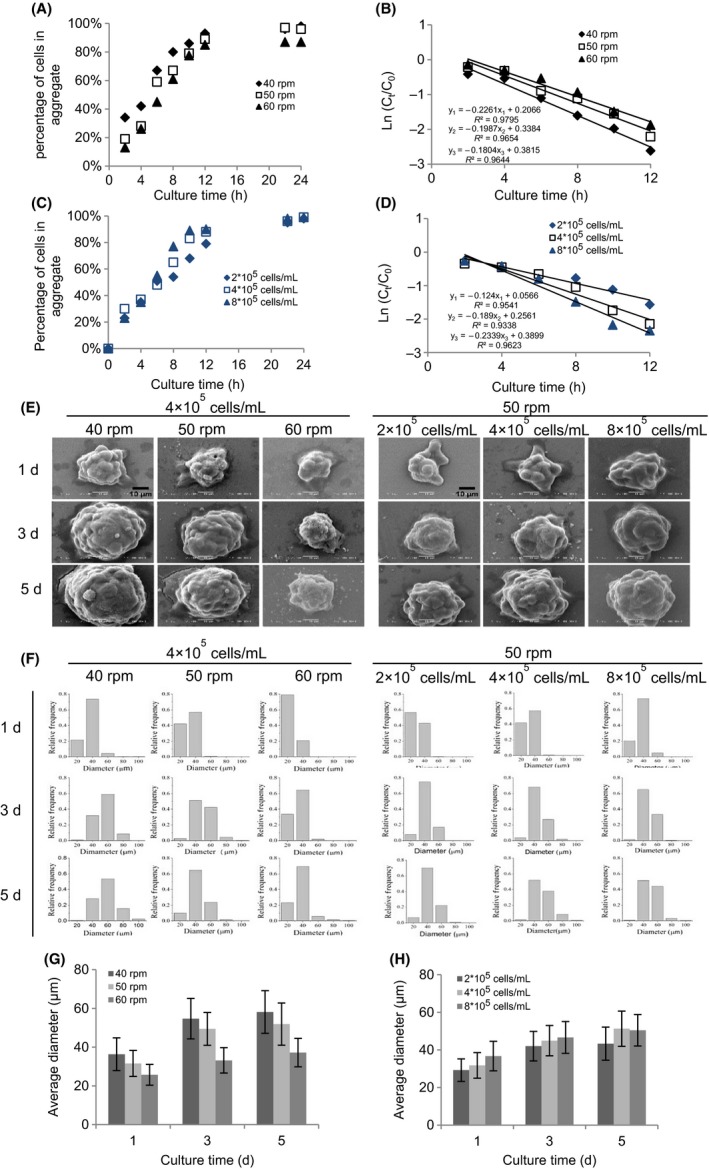
Formation of bACs aggregates in spinner flask. The effect of agitation rate (40, 50 and 60 rpm) on the (A) bACs aggregation and (B) kinetics of bACs aggregation over 24 h of culture. The effect of cell inoculum density (2, 4 and 8 × 10^5^ cells/mL) on the (C) bACs aggregation and (D) kinetics of bACs aggregation over 24 h of culture. (E) Low‐magnification (2000×) SEM analyses of bACs aggregation after 1, 3 and 5 d of culture. Scale bar = 10 μm. The size distribution (F) and the average size distribution (G, H) of bACs aggregates after 1, 3 and 5 d of culture (n = 500‐1000 per time point)

As shown in Figure 1A,C, bACs incorporated in aggregates were calculated by using the Equation (1), and the result showed that in all experimental conditions the percentage of bACs in aggregates increased with culture time. And nearly all the cells were encased in the aggregates at 24 hours. It was also found that with increased agitation rate, the rate of cellular aggregates decreased (Figure 1A). Initially, a higher percentage of bACs in aggregates in 4 × 10^5 ^cells/mL was observed at 50 rpm than those in 2 × 10^5^ and 8 × 10^5^ cells/mL (Figure 1C). This trend began to change from 6 hours, and from this time point till the end of culture, more chondrocytes aggregated at 8 × 10^5^ cells/mL. Based on the disappearance of single cells from suspension with time, the kinetics of cell aggregation was calculated by Equation (3). As seen in Figure 1B, the concentration of single bACs in suspension decreased exponentially with time, and the kinetic model fitted the data at 40, 50 and 60 rpm (*R* ^2^ > 0.95). bACs exhibited an increased *k* value at a lower agitation rate, with about 20% and 12% higher at 40 rpm than at 50 and 60 rpm, respectively. The formation of bACs aggregates at different cell densities also followed the first‐order kinetics, with *R* ^2^ > 0.90 at 4 × 10^5^ cells/mL and *R* ^2^ > 0.95 at 2 × 10^5^ cells/mL and 8 × 10^5^ cells/mL (Figure 1D). There were differences in the kinetics of bACs aggregation with respect to cell inoculation density. Higher *k* values were observed with an increase in cell inoculation density, with about 47% higher at 8 × 10^5^ cells/mL and 34% high at 4 × 10^5^ cells/mL than that at 2 × 10^5^ cells/mL for bACs. The results showed that agitation rate and cell inoculation density modulated cell aggregation and a lower agitation and higher cell inoculation density promoted the aggregation of bACs.

To monitor bACs aggregation, SEM was used to observe the surface characteristics of cellular aggregates after 1, 3 and 5 days of culture in spinner flask (Figure 1E). It was found that bACs formed compact and spherical aggregates on day 1, while the surfaces of aggregates were irregular. The size of cellular aggregates increased gradually with culture time, and at the same time, the surfaces became regular and smooth (Figure S1). In addition, a higher agitation rate and lower cell inoculation density decreased the size of cellular aggregates.

Based on image analysis of aggregate size, the size of bACs aggregates mainly ranged between 20 and 40 μm after 1 day, and the size distribution gradually became broad on day 3 and 5 (Figure 1F). From day 1 to day 5, while smaller aggregates turned less, larger ones strikingly increased in all culture conditions, indicating that the dynamic condition might promote the fusion of cellular aggregates. Moreover, the average diameter of bACs aggregates significantly increased with extended culture time, decreased agitation rate and increased cell inoculation density (Figure 1G,H).

### 3.2 Formation of rMSCs aggregates in spinner flask

**Figure 2:**
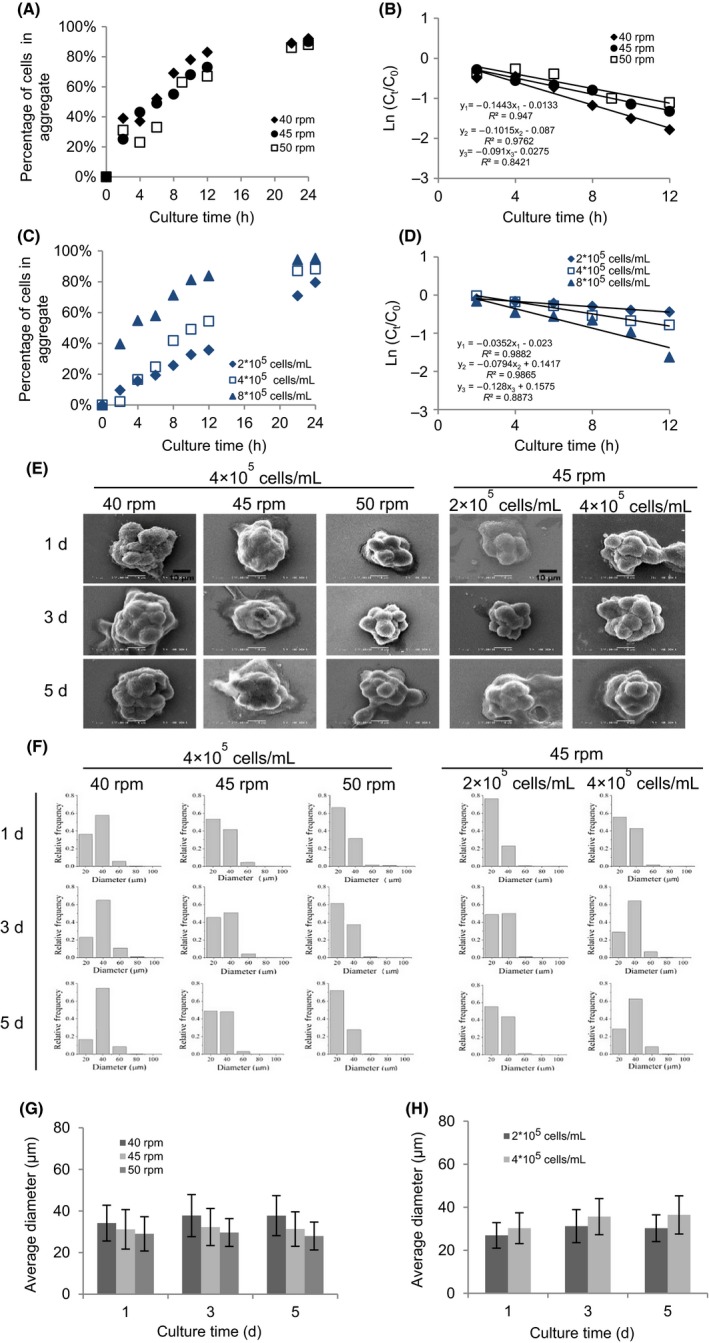
Formation of rMSCs aggregates in spinner flask. The effect of agitation rate (40, 45 and 50 rpm) on the (A) rMSCs aggregation and (B) kinetics of rMSCs aggregation over 24 h of culture. The effect of cell inoculum density (2, 4 and 8 × 10^5^ cells/mL) on the (C) rMSCs aggregation and (D) kinetics of rMSCs aggregation over 24 h of culture. (E) Low‐magnification (2000×) analyses of rMSCs aggregation after 1, 3 and 5 d of culture. Scale bar = 10 μm. The size distribution (F) and the average size distribution (G, H) of rMSCs aggregates (n = 500‐1000 per time point)

After 24 hours culture, in all experimental groups, about 80% of rMSCs were incorporated in cellular aggregates (Figure 2A,C). The rate of aggregates fluctuated before 6 hours, but consistently increased with extended culture time and decreased agitation rate, and increased cell seeding density afterwards. As seen in Figure 2B, the first‐order kinetic model fitted the disappearance of singles from the culture with time at 40 rpm (*R* ^2^ > 0.90), 45 rpm (*R* ^2^ > 0.90), and 50 rpm (*R* ^2 ^> 0.80) based on the Equation (3). For all acceptable correlations (*R* ^2 ^>0.80), rMSCs exhibited an increased aggregation rate with a lower agitation rate, and *k* was approximately 30% and 37% higher at 40 rpm than at 45 and 50 rpm. It was also found that rMSCs aggregation at different cell densities followed the simple first‐order kinetic model, with *R* ^2^ > 0.95 at 2 × 10^5^ and 4 × 10^5^ cells/mL and *R* ^2^ > 0.85 at 8 × 10^5^ cells/mL (Figure 2D). Taken together, this indicated that a lower agitation rate and higher cell inoculation density supported faster aggregation.

To monitor the aggregation of rMSCs, SEM was used to assess the surface characteristics of the aggregates after 1, 3 and 5 days of culture in spinner flask. As shown in Figure 2E, rMSCs formed spherical cell aggregates, which were different from the fusiform in 2D monolayer culture. An irregular topology of the aggregates was also observed during the culture process. Images at higher magnifications revealed that cellular aggregates showed no distinct gap between neighbouring cells (Figure S2). The size of rMSCs aggregates was decreased with a higher agitation rate and lower cell seeding density. When seeded at 8 × 10^5^ cells/mL, nearly all rMSCs aggregates settled to the bottom of spinner flask after 24 hours of culture, indicating that 45 rpm might not be sufficient for suspension culture of large aggregates (data not shown).

As for the size distribution of rMSCs aggregates, there was no obvious variation at 45 and 50 rpm throughout the culture, but the diameter slightly changed at 40 rpm after 5 days of culture (Figure 2F). The frequency of smaller rMSCs aggregates reduced with culture time at all different cell seeding densities, while larger aggregates became more. In the meantime, the percentage of larger aggregates was significantly higher at a low agitation rate and high cell inoculation density. The average diameter of rMSCs aggregates significantly increased with decreased agitation rate and increased cell seeding density (Figure 2G,H).

### 3.3 Molecular mechanism of cell aggregation

Cell adhesion molecules and ECM were important regulators of cell adhesion. To reveal the molecular mechanism of bACs and rMSCs aggregation in spinner flask, gene expression of cell adhesion molecules (cadherin and integrin β_1_) and ECM component (fibronectin) was analysed by qRT‐PCR and that of single cells before culture was used as control.

**Figure 3:**
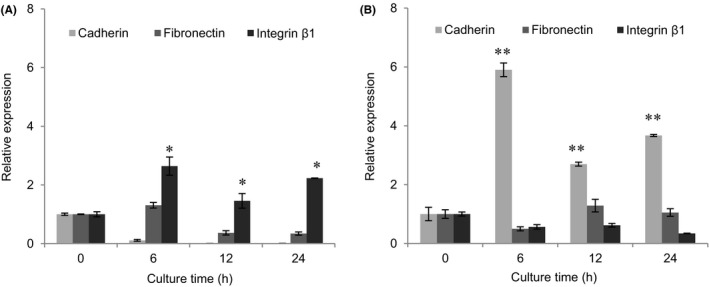
Molecular mechanism of cells aggregation. Expression of cell adhesion molecules (Cadherin and Integrin β_1_) and ECM component (Fibronectin) mRNA were analysed by qRT‐PCR within 24 h of culture. A, bACs aggregation is driven by integrin β_1_. B, rMSCs aggregation is driven by cadherin (n = 3). *P < 0.05, **P  < 0.01 compared to day 0

In the process of bACs aggregation, the levels of gene expression of cadherin and fibronectin were significantly lower than integrin β_1_ (Figure 3A). The expression of cadherin, a common cell adhesion molecule mediated cell‐cell interaction, was significantly downregulated with the time and nearly not detected after 12 hours. bACs cultured in spinner flask showed reduction in gene expression of fibronectin in contrast with control group. Gene expression of integrin β_1_, a cell adhesion molecule mediated cell adhesion to ECM, was significantly unregulated from 0 to 6 hours in spinner culture, and maintained the high expression level from 6 to 24 hours, which was in consistent with the kinetics of bACs aggregation.

During the formation process of rMSCs aggregates, the level of gene expression of cadherin was significantly higher than fibronectin and integrin β_1_ (Figure 3B). There was no obvious change in gene expression of fibronectin within 24 hours. Integrin β_1_ exhibited decreased gene expression in comparison with positive control. Cadherin of rMSCs in spinner flask kept high gene expression level throughout the culture period, similar to the kinetics of rMSCs aggregation.

### 3.4 Formation of mixed co‐aggregates of bACs and rMSCs in spinner flask

**Figure 4:**
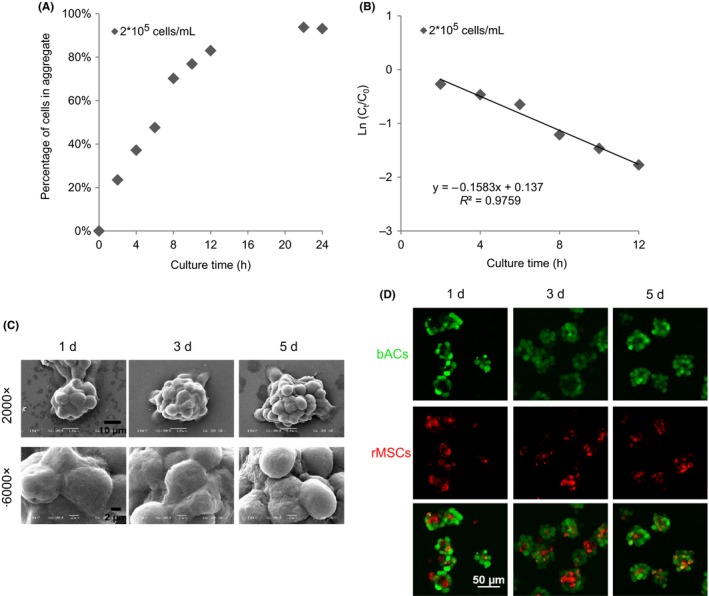
Formation of mixed co‐aggregates of bACs and rMSCs in spinner flask. (A) bACs and rMSCs aggregation and (B) kinetics of bACs and rMSCs aggregation over 24 h of culture. (C) Low‐magnification (2000×, Scale bar = 10 μm) and high‐magnification (6000×, Scale bar = 2 μm) analyses of mixed co‐aggregates after 1, 3 and 5 d of culture. (D) Confocal microscopy analyses the location and distribution of mixed co‐aggregates composed of bACs and rMSCs. Scale bar = 50 μm

Similar to respective culture of bACs and rMSCs, coculture of bACs and rMSCs in spinner flask also formed aggregates in 1 day. The amount of cells in aggregates increased with culture time, and over 95% were entrapped after 24 hours (Figure 4A). Similarly, the first‐order kinetic model fitted the aggregation of cocultured cells with time (*R* ^2^ > 0.95; Figure 4B).

By using SEM, an irregular topology of aggregates was identified on day 1 and to a greater extent on day 3 and 5 (Figure 4C). Images of higher magnification indicated that the surfaces of aggregates became progressively rough. The size of aggregates also increased with culture time. To investigate cell distribution in the aggregates, confocal microscopy was adopted to display cell morphology. As seen in Figure 4D, both bACs and rMSCs were present in all mixed co‐aggregates. Cellular aggregates were relatively small, irregular and loose after 1 day, and then became larger and more compact with extended culture time. Images of high magnification clearly showed that bACs overlaid around rMSCs in the aggregates.

## 4 DISCUSSION

Cellular aggregates have been used in cell‐based therapy for cartilage tissue regeneration.^17^, ^18^ It has been documented that chondrocyte aggregates promote cell differentiation, secretion of ECM molecules and seeding onto scaffolds than monodispersed cells.^19^ MSCs can be induced to undergo chondrogenic differentiation in vitro via cellular aggregation which essentially mimics MSCs condensation, a vital stage in the skeletal development.^20^ Therefore, it is worth exploring cellular aggregates for cartilage regeneration. Culture in spinner flask has been reported to induce the aggregation of both bACs and rMSCs readily.^4^, ^15^ In dynamic 3D environment, the agitation rate and initial cell density are implicated to be very critical in modulating cell‐cell and cell‐aggregate contacts and then the formation of cellular aggregates.^21^

In the present study, the aggregation of bACs, rMSCs and their mixture in different culture conditions was characterized. Particularly, the use of cells from different species was mainly for conveniently distinguishing between two cell types in subsequent coculture studies, specifically for genetic analysis, albeit it is not the main focus of the present study. It was demonstrated that both cell seeding density and agitation rate could affect the aggregation kinetics and the aggregate size. In the present study, the lowest agitation rate was set at 40 rpm, because cells would settle at the bottom of spinner flask within 1 day at lower agitation rates. By counting free single cells in the suspension, it was found that the percentage of cells in aggregates increased with extended time, decreased agitation and increased cell inoculation density. Bueno et.al examined the aggregation rate of bACs under different stirring speeds, and showed that bACs aggregates increased with time and the aggregation rate at 80 rpm was significantly lower than that at 50 rpm.^22^ Others also found that the aggregation rate increased with cell density.^16^ The aggregation of bACs, rMSCs and the mixture within 12 hours was well described by using a first‐order kinetic model. The low agitation rate and high cell seeding density promoted the aggregation of both bACs and rMSCs (higher *k* values), because the higher agitation rate caused a greater shear force, which partially offset the adhesive strength between cells, leading to the disintegration of cellular aggregates.^23^ Thus, the agitation rate is not only responsible for cell aggregation, but also their destruction. Meanwhile, a higher cell seeding density resulted in an increase in the collision frequency between cells, and cells and the aggregates, which could promote the formation of cellular aggregates. Previously, Portanin et al^24^ had shown that the first‐order kinetics of cell aggregation could be described as the coagulation of particles in shear flow, and the aggregation was linearly related to cell density. These observations showed that the kinetics of cell aggregation could be affected by the agitation rate and cell inoculation density.

The size of chondrocyte aggregates can be important in their application in cartilage regeneration. One previous study showed that cellular aggregates at a micrometer scale produced greater collagen II and GAG than macrometer‐sized (1‐2 mm) aggregates.^25^ Cellular aggregates with a smaller diameter can reduce the diffusion gradients of nutrients for growth and result in more uniform cell behaviours. Moreover, another study reported that chondrocytes aggregates with a diameter of 20‐32 μm enhanced the cell attachment on polyglycolic acid scaffolds with 97% porosity.^19^ Similarly, the size of MSCs aggregates can also affect their differentiation potential. MSCs aggregates with a smaller dimension tended to form more homogeneous chondrogenic differentiation towards cartilage whereas the larger aggregates produced the heterogeneous tissue.^26^ In the present study, the average diameters of chondrocyte and MSCs aggregates were within the range of 20‐60 and 20‐40 μm, respectively. The majority of the cells were viable in aggregates after 5 days of culture in spinner flask (data not shown). Both bACs and rMSCs exhibited the larger average diameter with lower agitation rate and higher cell seeding density. Vigorous agitation would induce the instability of large cellular aggregates, leading to the generation of small aggregates and reduction in the apparent size of cellular aggregates.^27^ On the other hand, less intense agitation reduces the frequency of collision, but enhances the fusion efficiency of cells.^23^ In addition, a higher cell seeding density accelerates the collision frequency between cells and also enhances the formation of aggregates. The formation of cell aggregates from single cells was mainly occurred within 1 day. Then, the average diameter of the aggregates increased with culture time, especially chondrocytes aggregates. In the meantime, the percentage of aggregates decreased with culture time, especially in the first 3 days, which indicated the fusion of small aggregates into large ones. With the increased dimension of cellular aggregates, shear force and growing viscous resistance prevented the fusion of aggregates. bACs formed compact aggregates with larger dimension more readily than rMSCs due to the fact that bACs can secrete abundant extracellular matrix,^28^ which can potentially promote cell aggregation. These results demonstrate that the size of cellular aggregates can be controlled by agitation rate, cell seeding density and culture time.

The process of mesenchymal chondroprogenic cell condensations is driven by signals initiated by cell‐cell and cell‐ECM interactions.^20^ In the present study, it was demonstrated that direct cell‐cell interactions mediated by N‐cadherin were involved in rMSCs aggregation, while bACs aggregation depended on integrin β_1_, which mediates the interactions between cells and ECM. N‐cadherin is known to be required to initiate chondrogenesis of MSCs, and not expressed in differentiated chondrocytes.^29^ In mature hyaline cartilage, there is abundant ECM, which favours cell‐ECM interactions rather than cell‐cell contacts. However, fibronectin might not contribute to chondrocytes‐ECM interactions during cell aggregation, since gene expression of fibronectin had no obvious change. Instead, there was correlation between gene expression of collagen II and integrin β_1 _(Figure S3A). In fact, a previous study also implicated that the formation of chondrocytes aggregates in suspension culture was mainly through binding of collagen II with integrin β_1_.^30^

Coculture of MSCs and chondrocytes has been recognized a very promising strategy for cartilage regeneration.^31^ In the present study, aggregates formed with a mixture of bACs and rMSCs were also successfully prepared, and the first‐order kinetic model was able to well describe the aggregation process of the mixture. Moreover, it is very interesting to find under confocal microscopy that the mixture of bACs and rMSCs (the number ratio at 1:1) generated core‐shell structured cellular aggregates, wherein bACs covered rMSCs tightly. It had been implicated that cells releasing more cadherin could help to migrate to the interior of the aggregates.^32^ Steinberg guessed that cell‐to‐cell adhesion and cell sorting behaviour in embryonic tissues are the need of maximized adhesion and minimized energy.^33^ The adhesion molecules regulate cell surface tension which cells released more cadherin forming the core of the aggregates and those cells produced high integrin forming the periphery.^34^ In the present study, the molecular mechanisms of cell aggregation, the expression level of cadherin and integrin β_1_ have been tested. The consistent tendency was also found which was consistent with former report. Certainly, mechanisms behind the morphology form different cell type were more complex than described, which still need more investigation.

In summary, the present study demonstrated that spinner flasks bioreactor provided dynamic 3D environment which induces the numerous aggregation of bACs, rMSCs and mixture of this two cells, respectively. These aggregates can be directly used in cell‐based therapies. The key culture parameters to the aggregation process, and the kinetics and molecular mechanism of aggregation were identified. The rate of aggregation and the size could be effectively controlled by various applications. Further observation revealed that chondrocyte phenotype and stemness of MSCs (Figure S3) were preserved in aggregates. This would serve as foundation for large‐scale production of cellular aggregates for cartilage regeneration.

## CONFLICT OF INTEREST

The authors declare no commercial or financial conflict of interest.

## AUTHOR CONTRIBUTIONS

Zhaoyang Ye and Yan Zhou designed research; Huimin He and Qing He performed experiments; Huimin He, Zhaoyang Ye, Yan Zhou and Wen‐Song Tan analysed data; and Feiyue Xu, Zhaoyang Ye, Yan Zhou and Wen‐Song Tan wrote the paper.
