# Supplementary material for: Dynamic formation of cellular aggregates of chondrocytes and mesenchymal stem cells in spinner flask
Source: Cell Prolif. 2019 Jun 17;52(4):e12587. doi: 10.1111/cpr.12587 (PMC6669002; doi:10.1111/cpr.12587)
Supplement: Supplementary file 1 [file CPR-52-e12587-s001.docx]

| **Bovine Gene** | **Primer sequences** |
| --- | --- |
| *GAPDH* | *Forward: 5，- GATGCTGGTGCTGAGTATGT -3，*  *Reverse: 5，- GCAGAAGGTGCAGAGATGAT -3，* |
| *Cadherin* | Forward: 5^，^- GGTCCTTGAGCTCCCTTAATTC -3  Reverse: 5^，^- CGTACATGTCAGCGAGTTTCT -3 |
| *Integrin β_1_* | Forward: 5^，^-AGGCCACTGTTCATGTTGTAG -3  Reverse: 5^，^-CAGCAATGCAAGGCCAATAAG -3 |
| *Fibronectin* | Forward: 5^，^- CCCACTCCTACAACCAGTATTC -3  Reverse: 5^，^- CTTCTCTGTCAGCCTGTACATC -3 |
| *Collagen II* | Forward: 5^，^- TGGCTTCCACTTCAGCTATG -3^，^  Reverse: 5^，^- CAGTGGTAGGTGATGTTCTGAG -3^，^ |
| *SOX-9* | Forward: 5^，^- TCTACACACAGCTCACCAGA -3^，^  Reverse: 5^，^- CGTTTGTTTCTTGGGTTCCTTC -3^，^ |
| *Aggrecan* | Forward: 5^，^- GAGTGGCAGTGGTGAATCTT -3^，^  Reverse: 5^，^- CCACAGATCCTAAGCCTTCTTC -3 |

**Supporting Information**

**Table S1**. Primer sequences for bovine genes. The expression levels of

mRNA were normalized to GAPDH.

| **Rabbit Gene** | **Primer sequences** |
| --- | --- |
| *GAPDH* | Forward: 5^，^- GCGTGAACCACGAGAAGTAT -3^，^  Reverse: 5^，^- CCTCCACAATGCCGAAGT -3^，^ |
| *Cadherin* | Forward: 5^，^- GGATGCAGATGATCCTACCTATG -3  Reverse: 5^，^- GAAGGGCTGTCCTGATGATAC -3 |
| *Integrin β_1_* | Forward: 5^，^-CCATCGACCTCTACTACCTCAT -3  Reverse: 5^，^-TGAAGTCACCCTCCTCATCT -3 |
| *Fibronectin* | Forward: 5^，^- AGCTGGGAGAGGGAGAATTA -3  Reverse: 5^，^- GCCTAGACACACACACTCTAAC -3 |
| *SOX-2* | Forward: 5^，^- ATGCACAACTCGGAGATCAG -3^，^  Reverse: 5^，^- TTTATAATCCGGGTGCTCCTTC -3^，^ |
| *OCT-4* | Forward: 5^，^- AAGGAGAAGCTGGAGCAAAC -3^，^  Reverse: 5^，^- TGAGTGTAGCCCAGAGTGAT -3^，^ |
| *Nanog* | Forward: 5^，^- GACAGAAATACCTCAGCCTTCA -3^，^  Reverse: 5^，^- TCTCTGCCACCTCTTACATTTC -3^，^ |

**Table S2**. Primer sequences for rabbit genes. The expression levels of

mRNA were normalized to GAPDH.


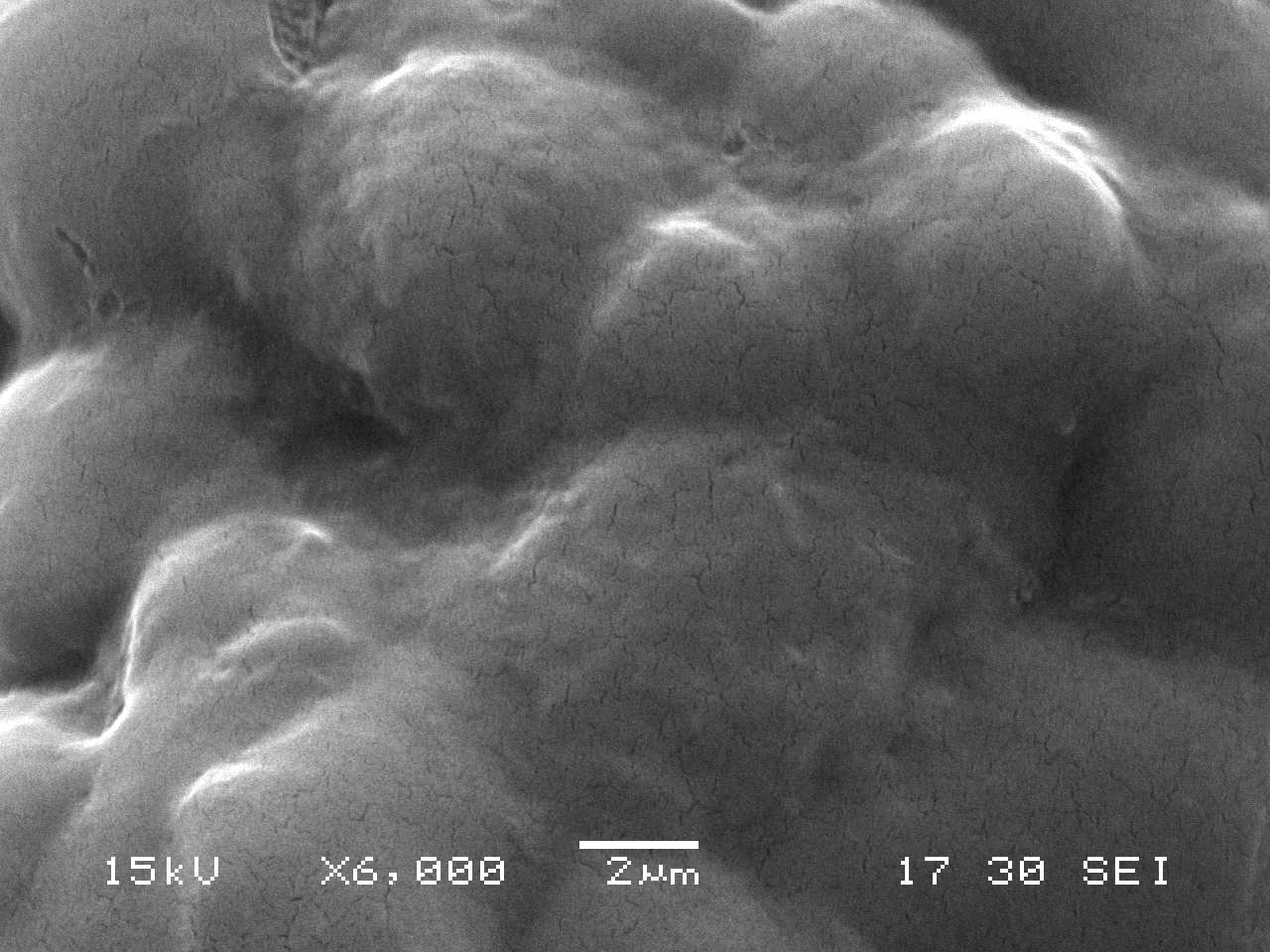

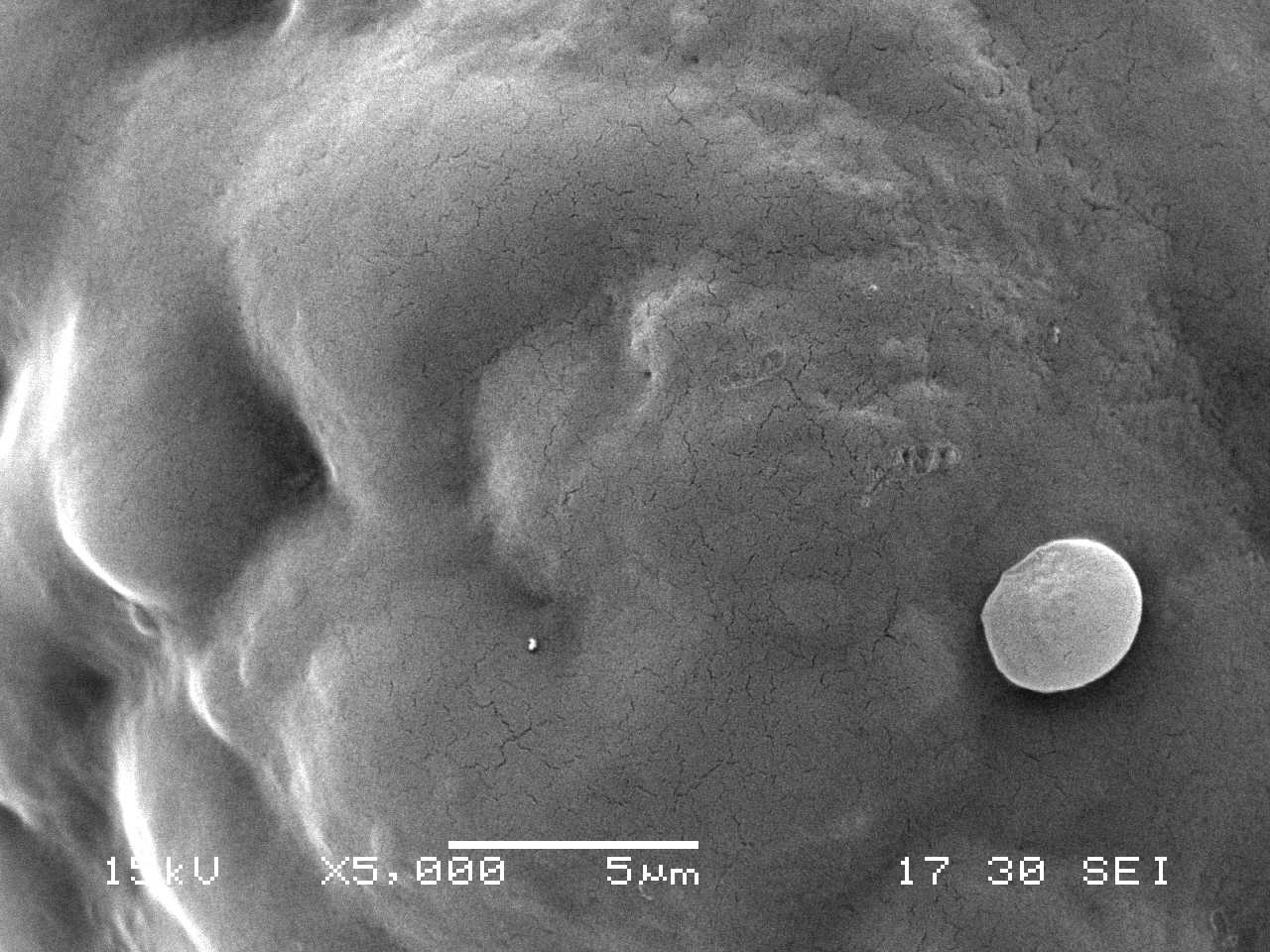

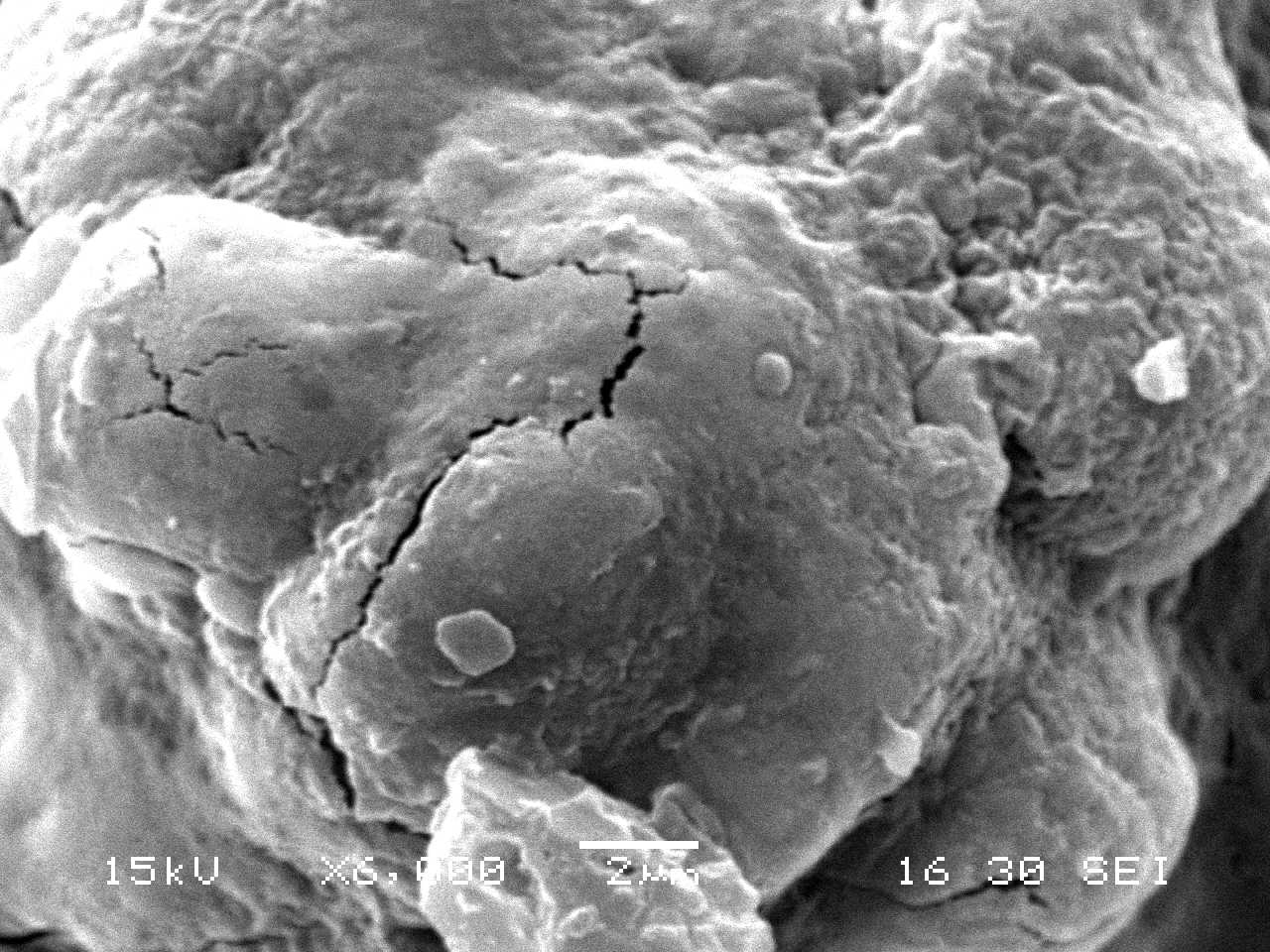

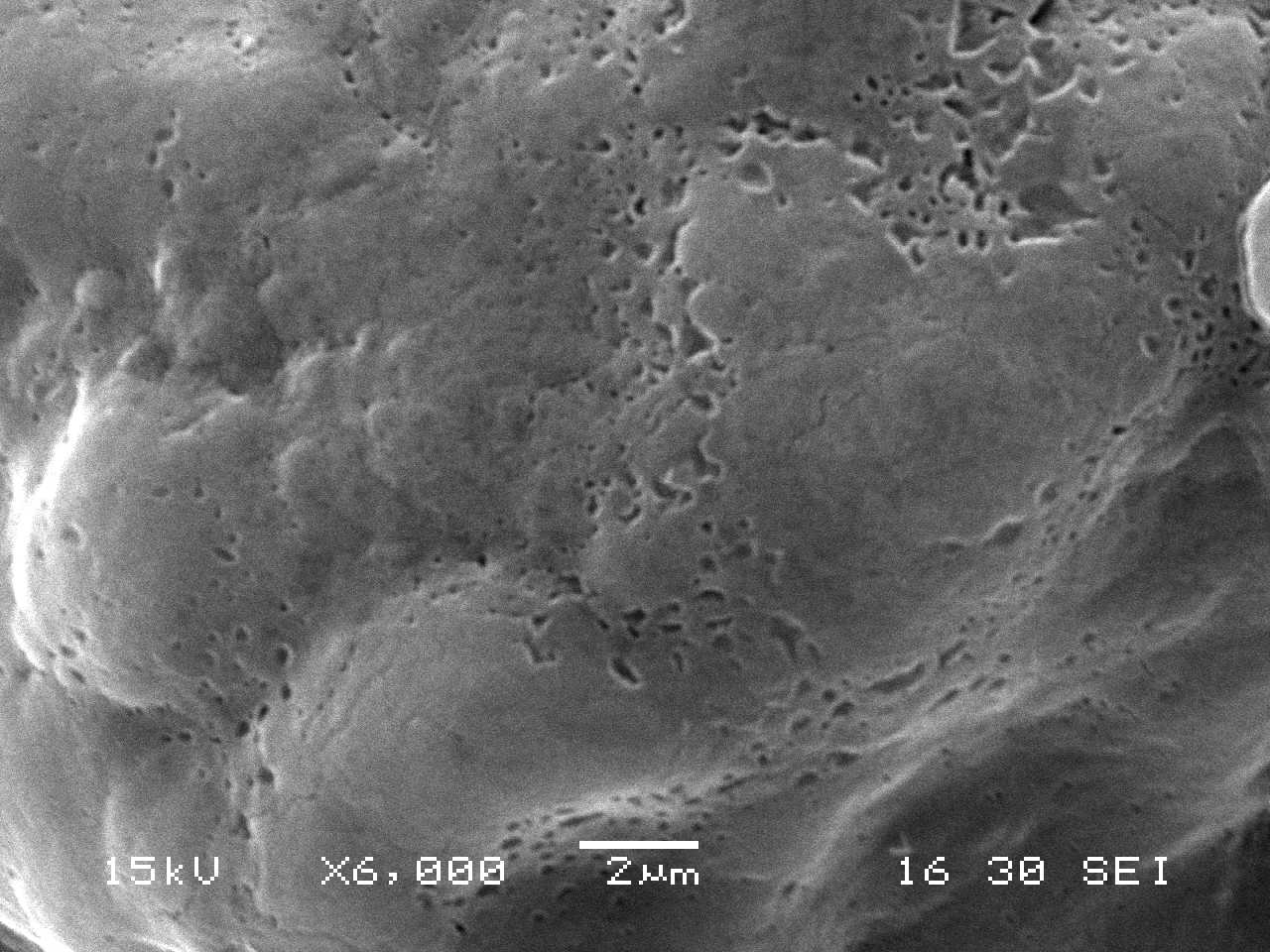

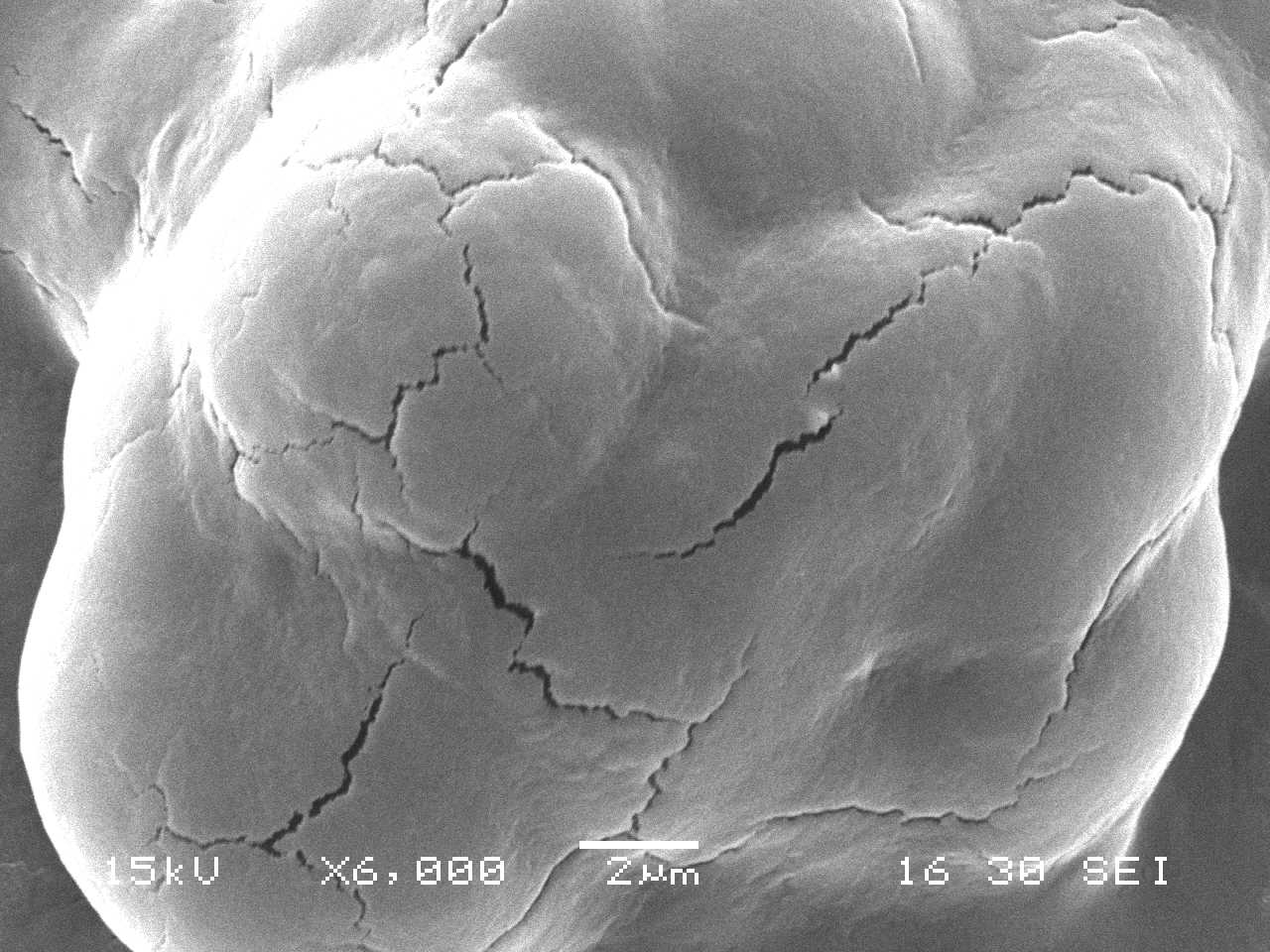


40 rpm

50 rpm

60 rpm


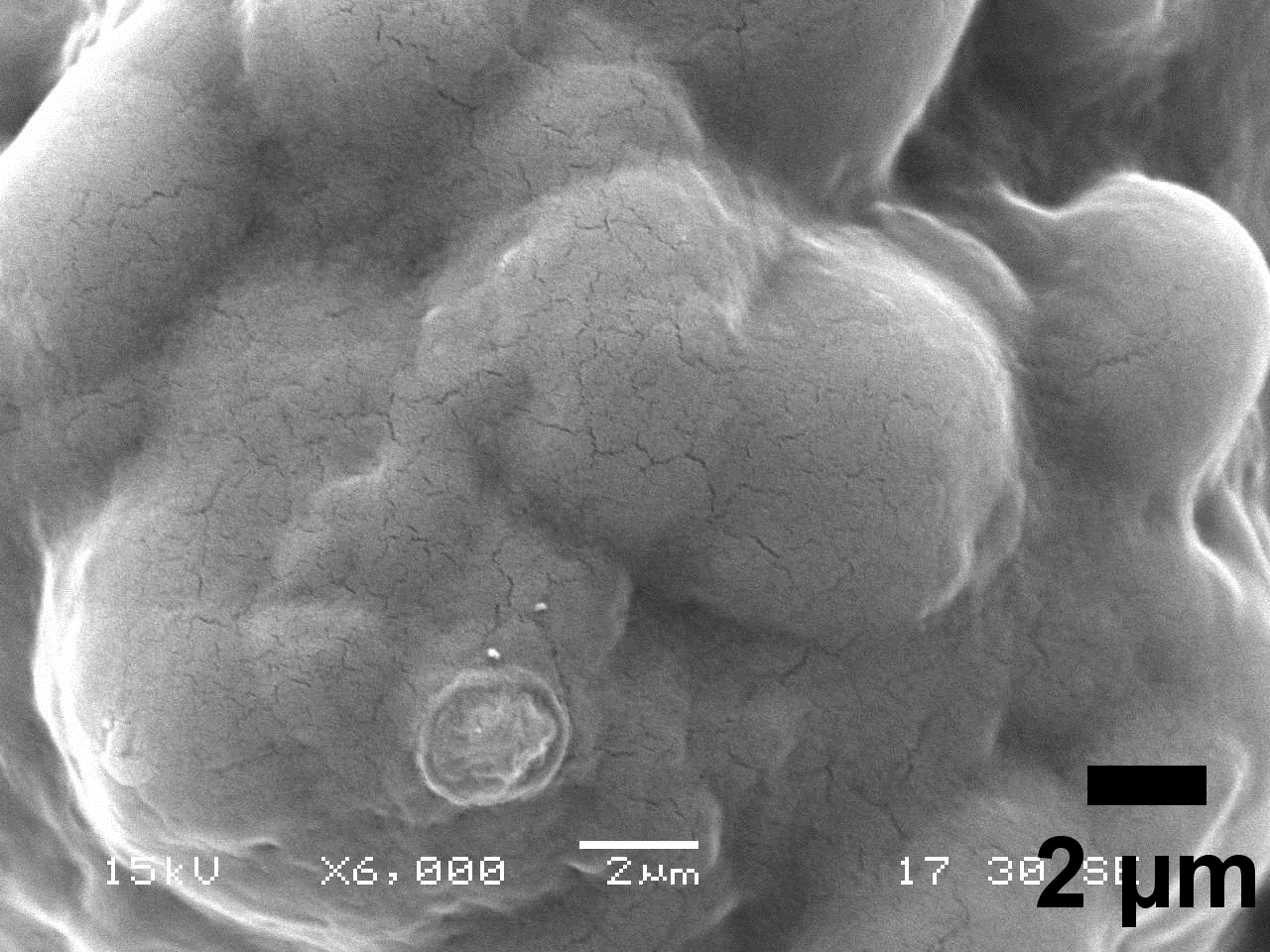

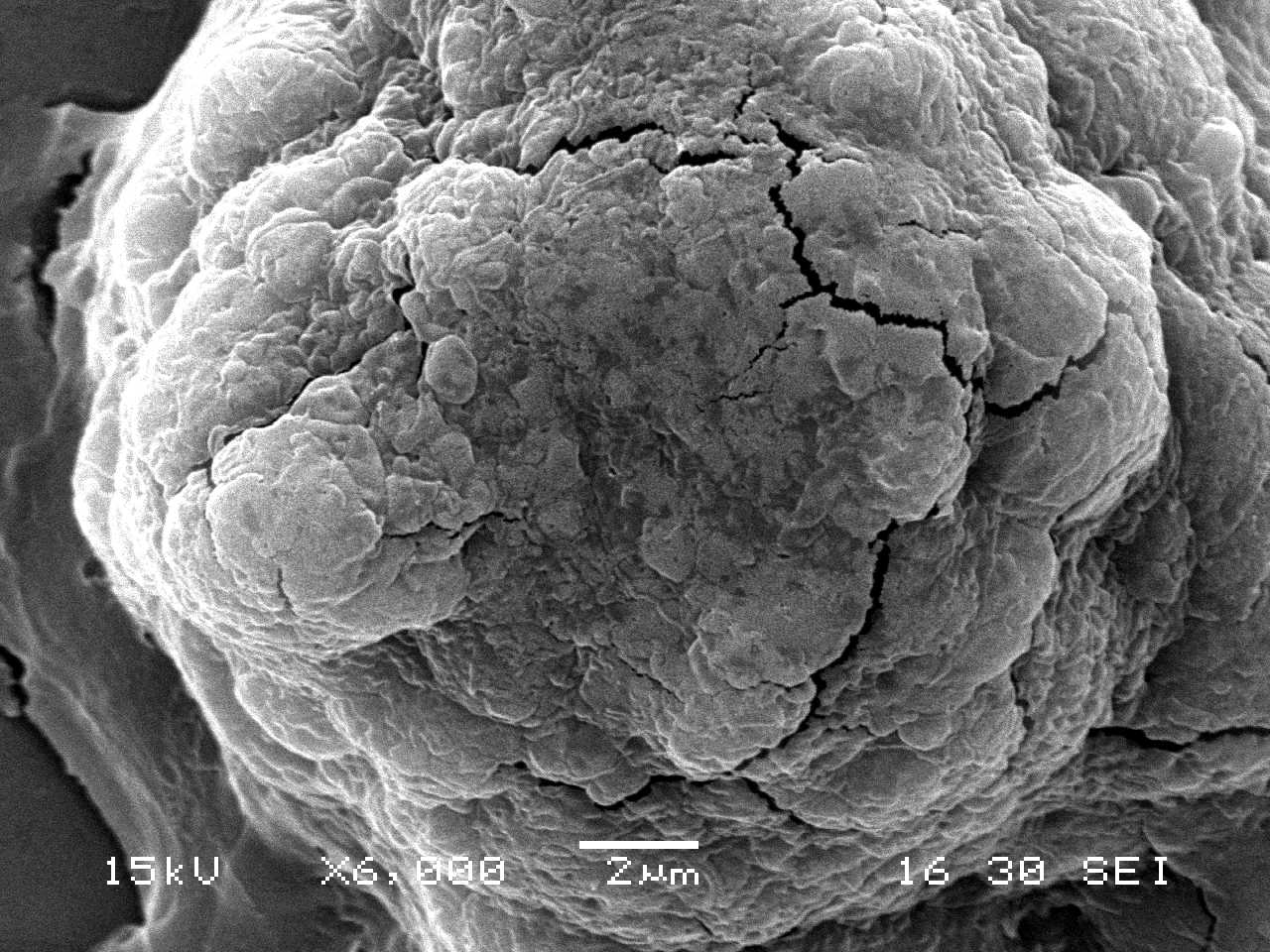

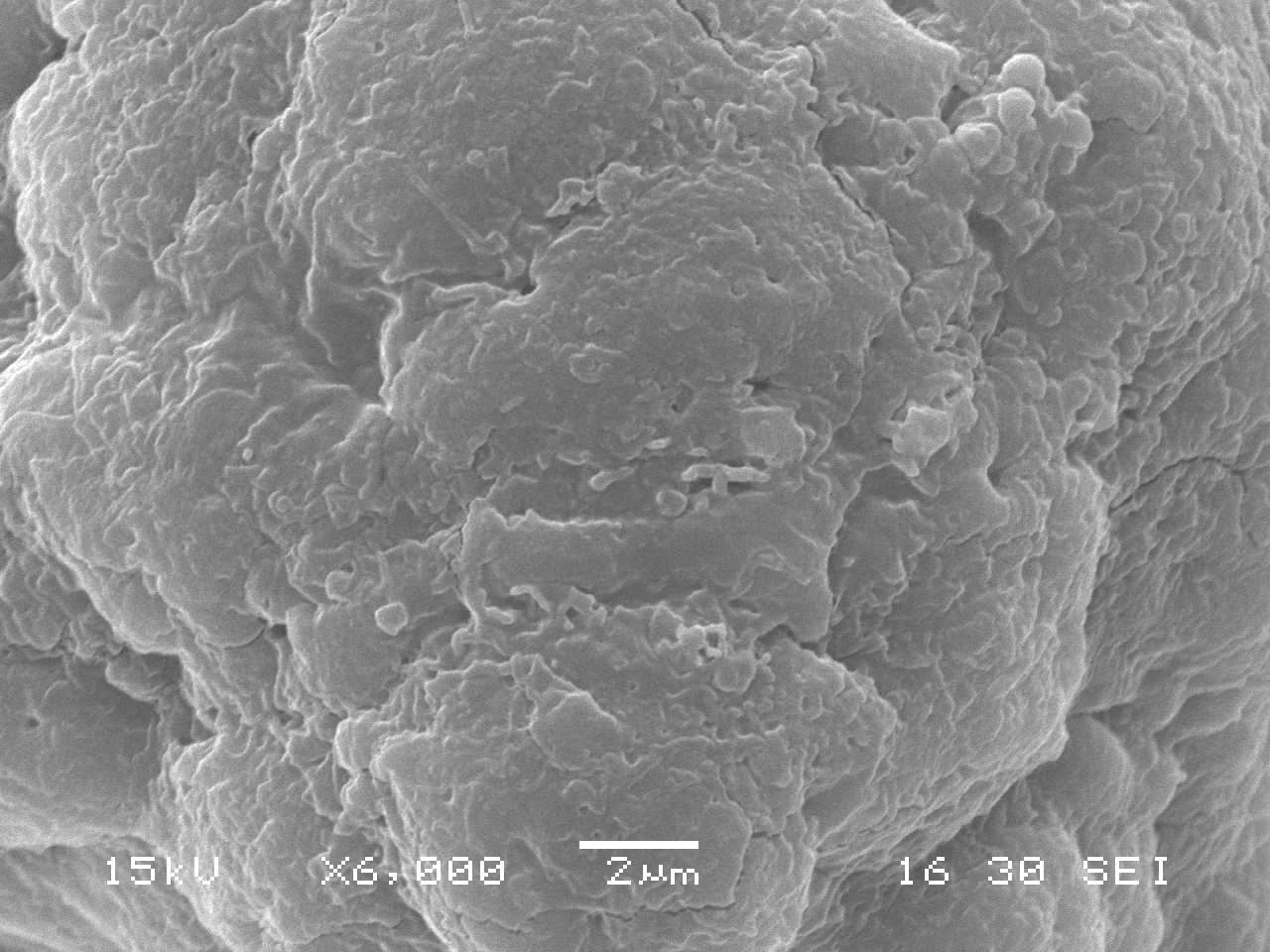


1 d

3 d

5 d


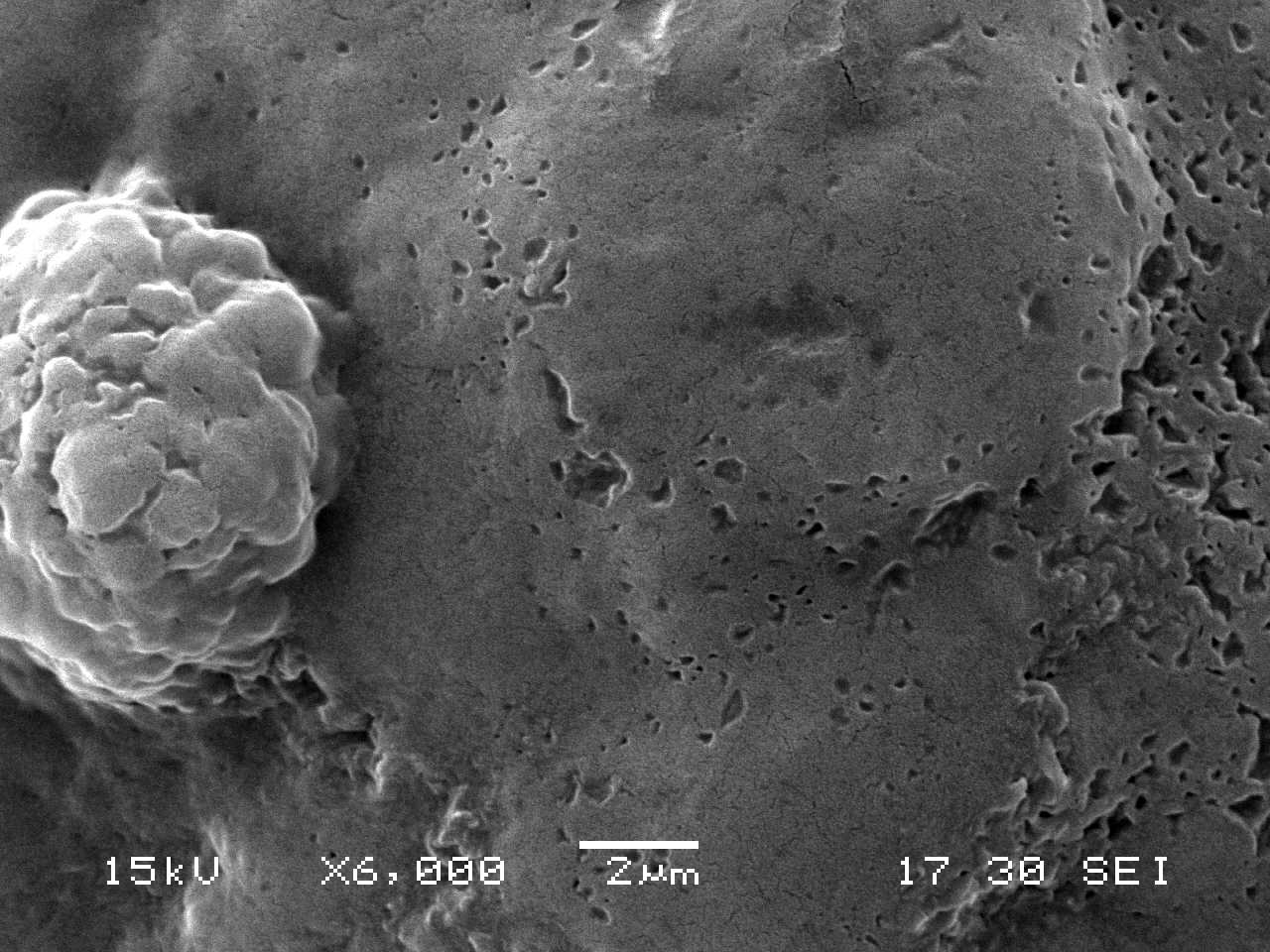

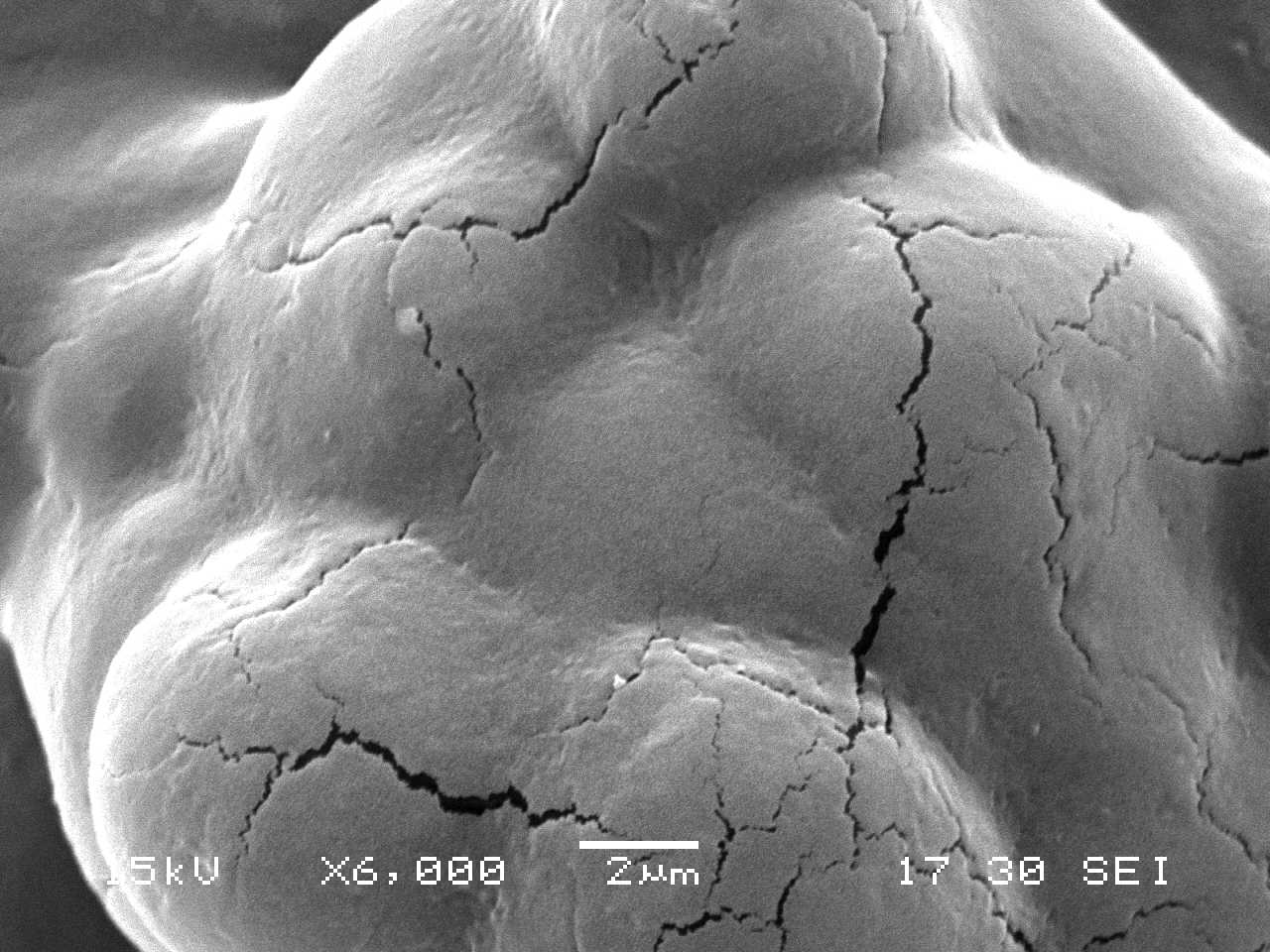

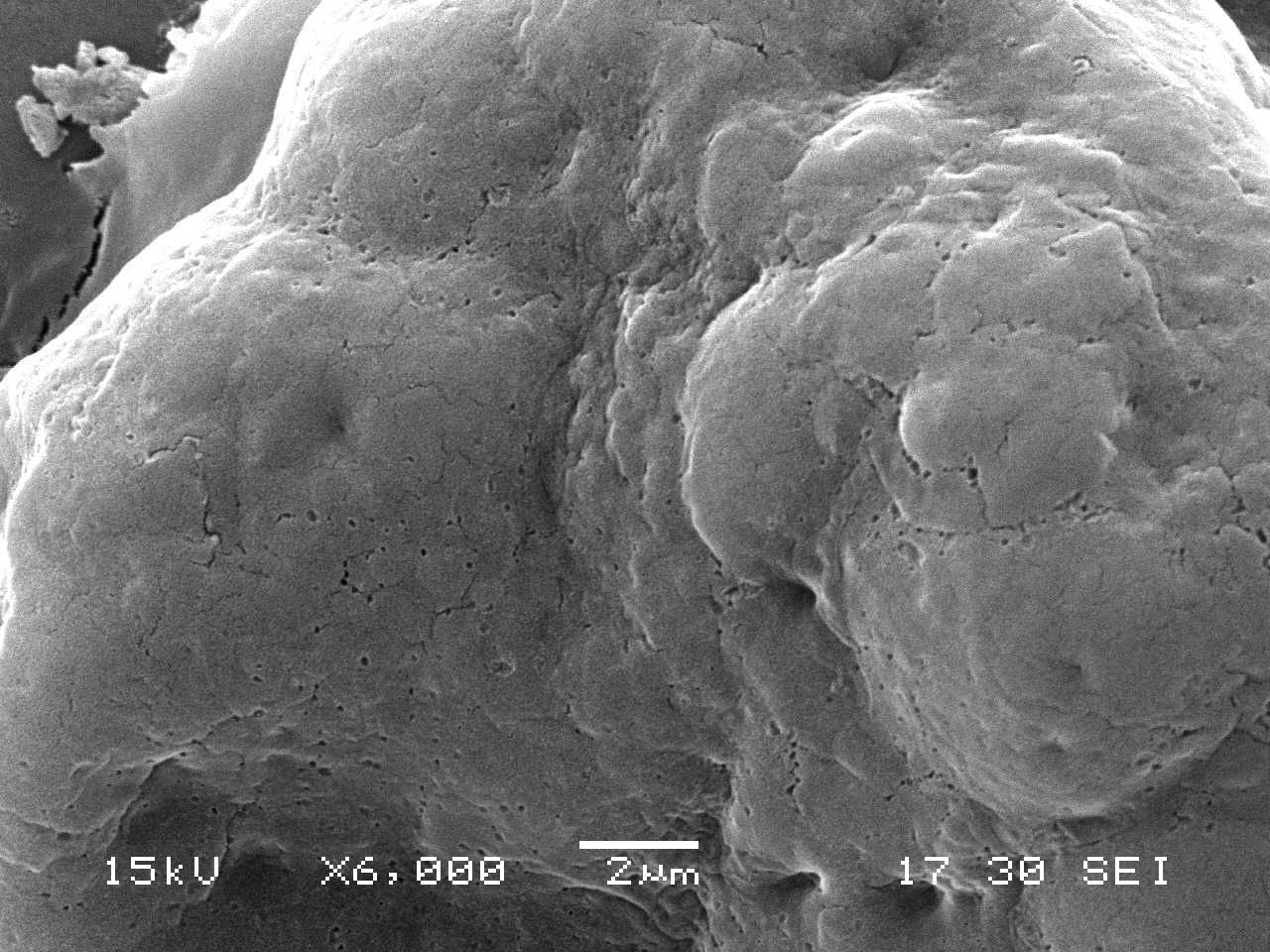

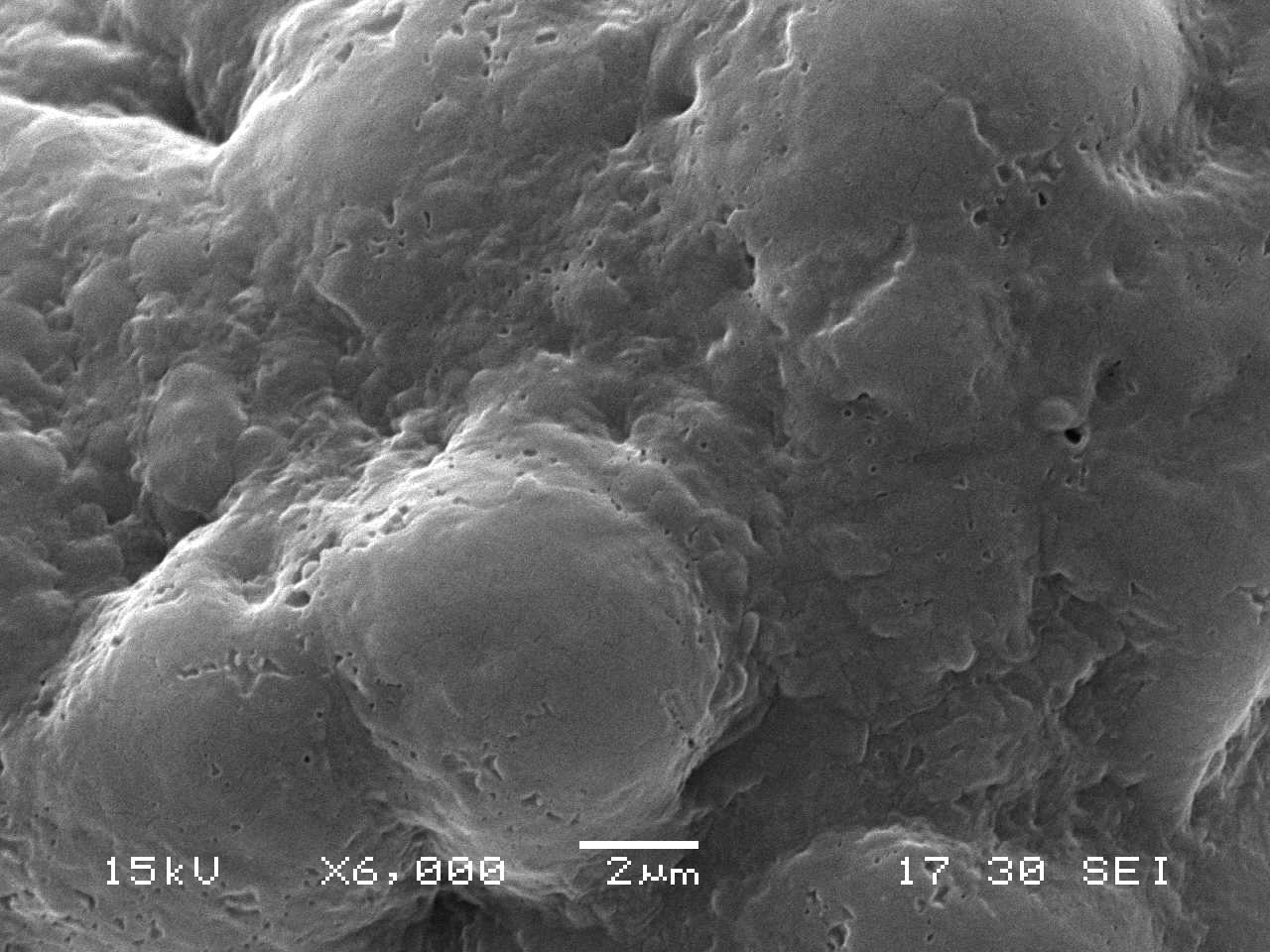

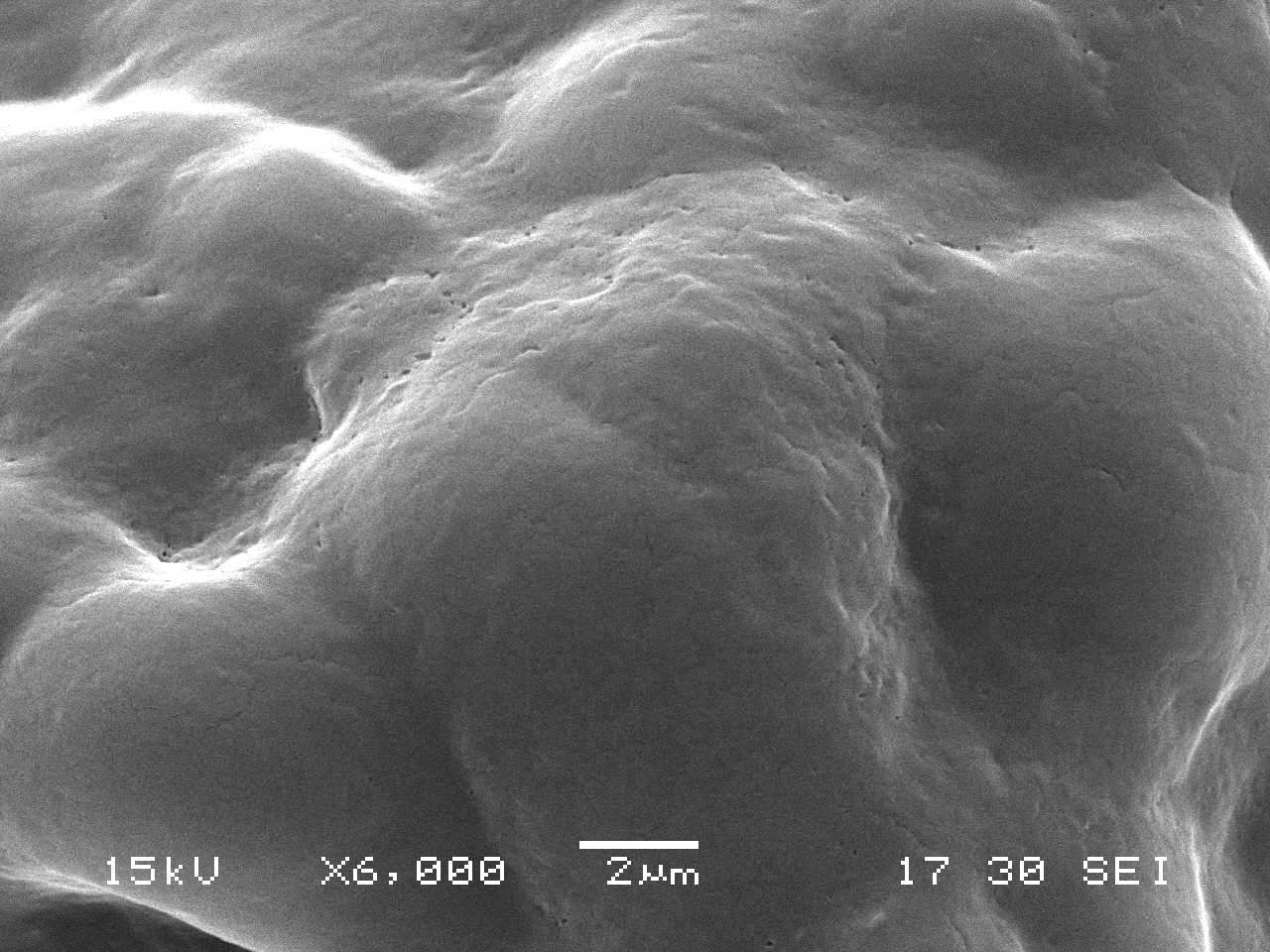

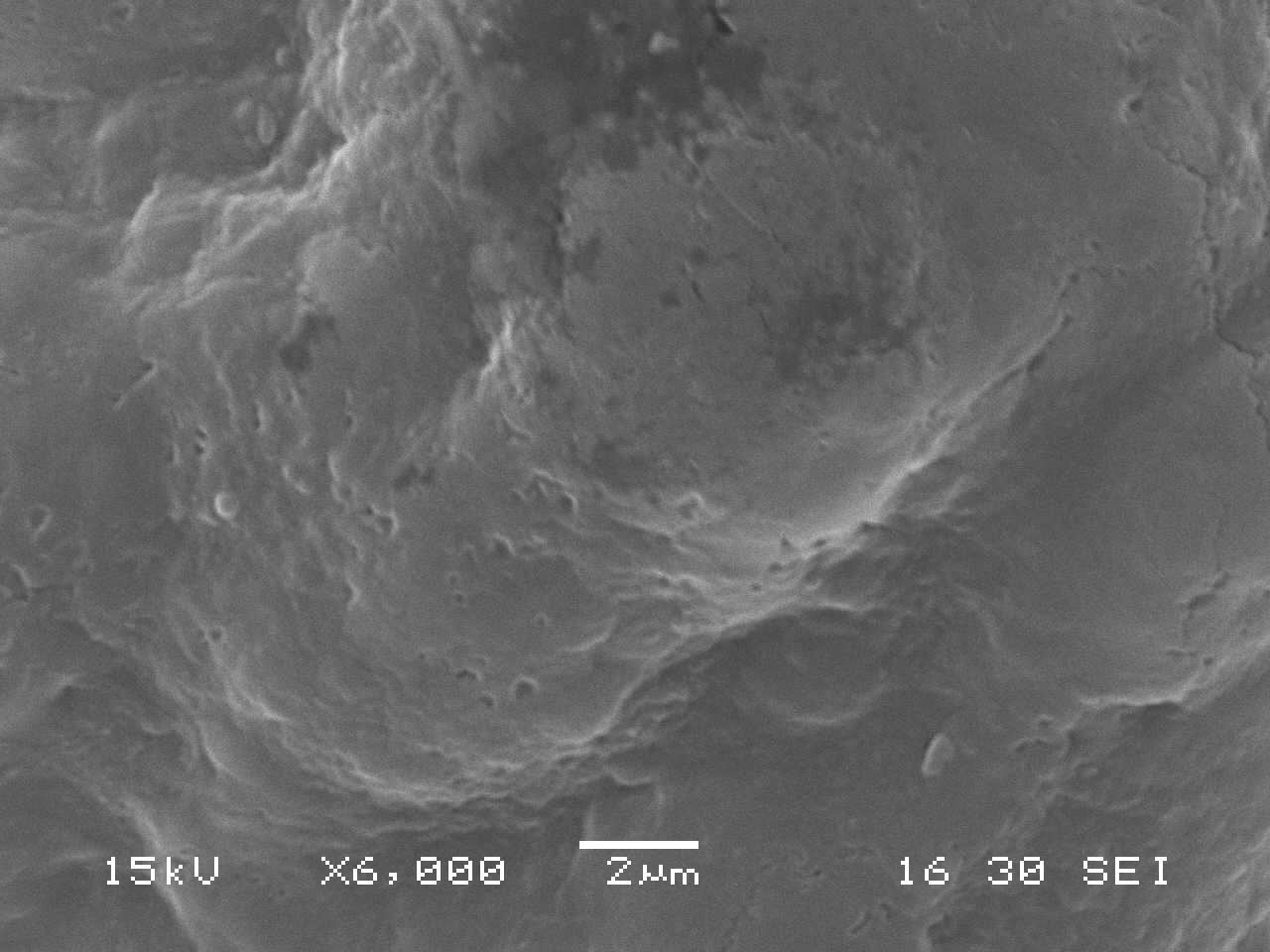

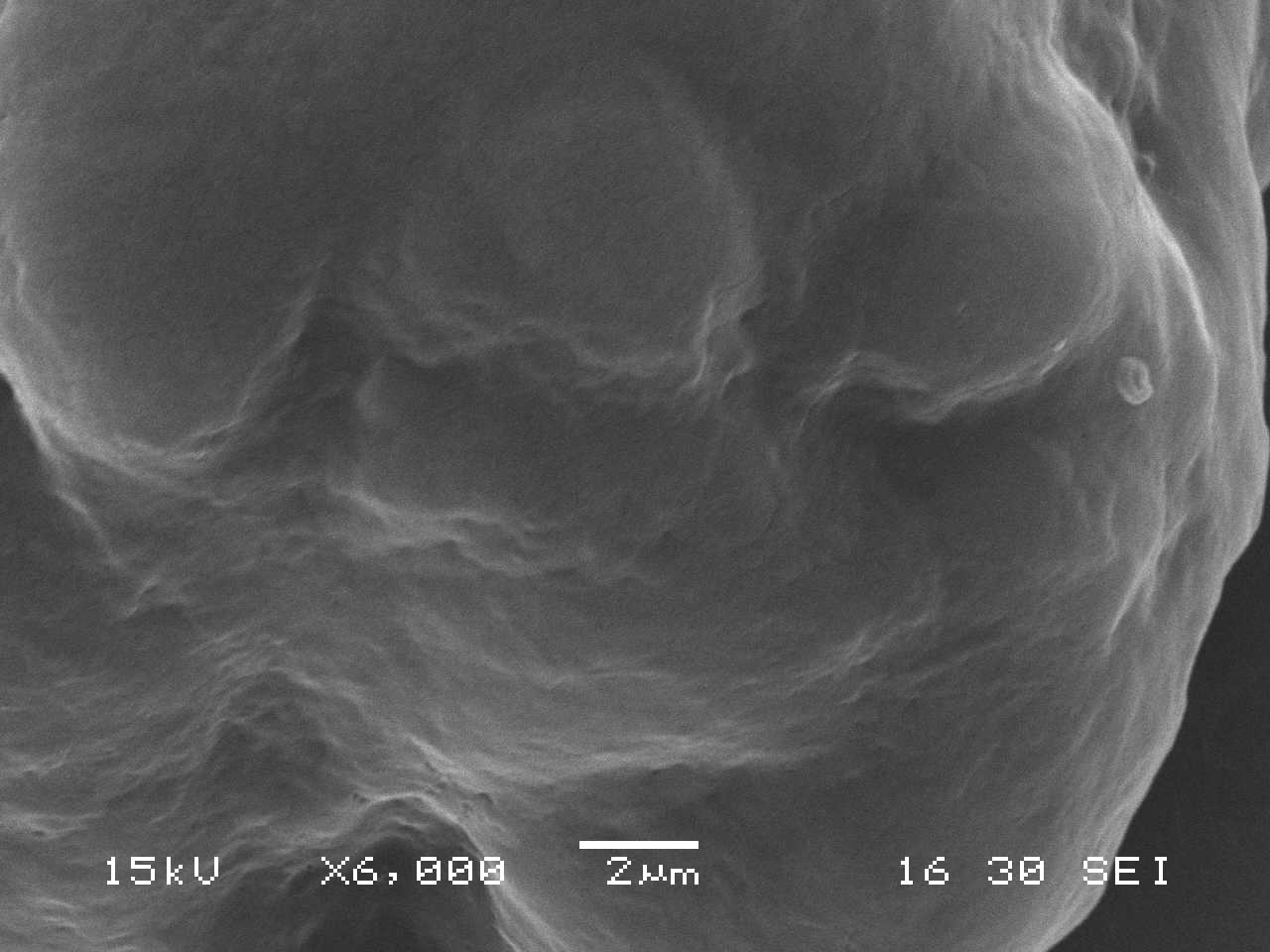

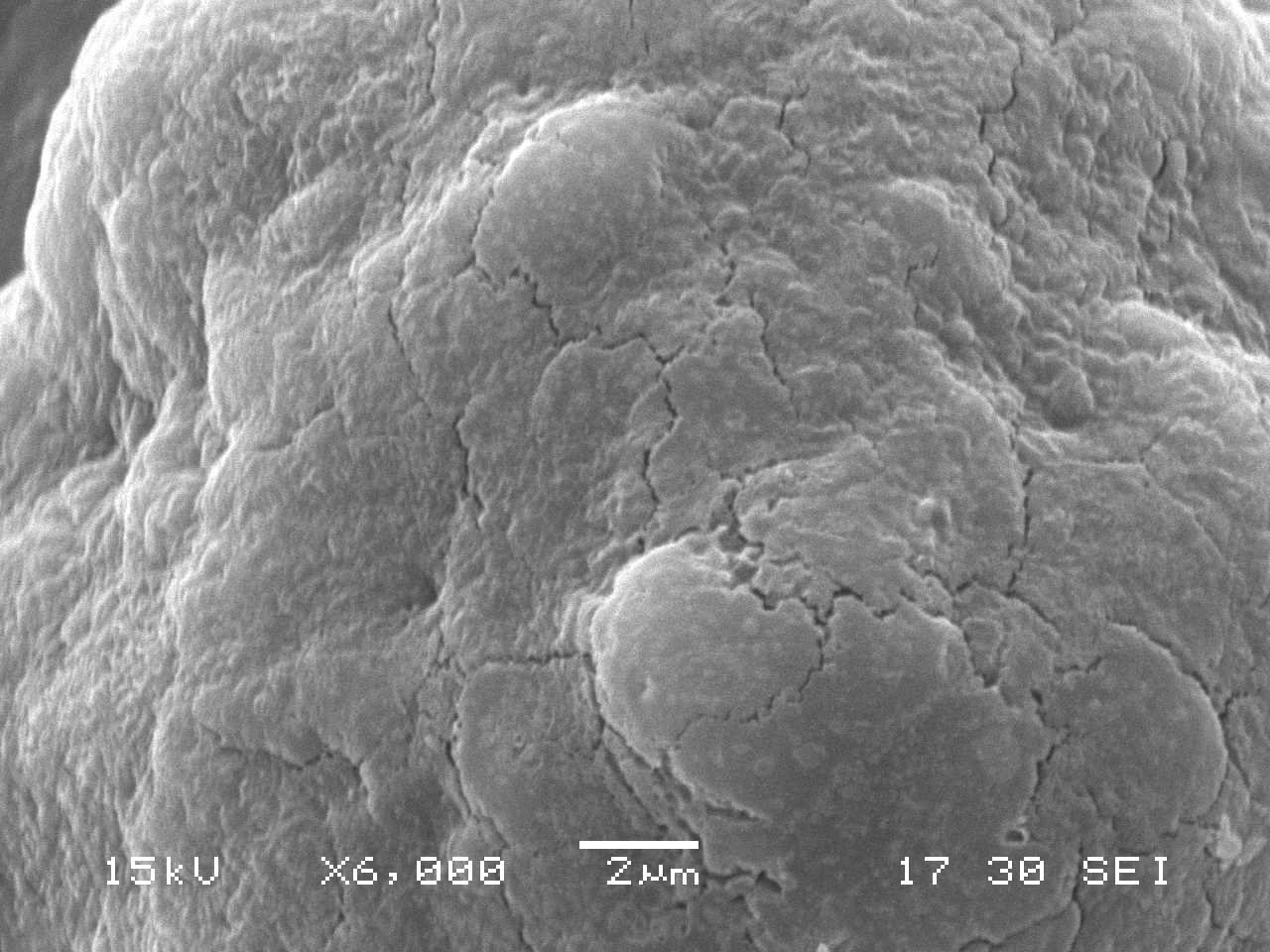

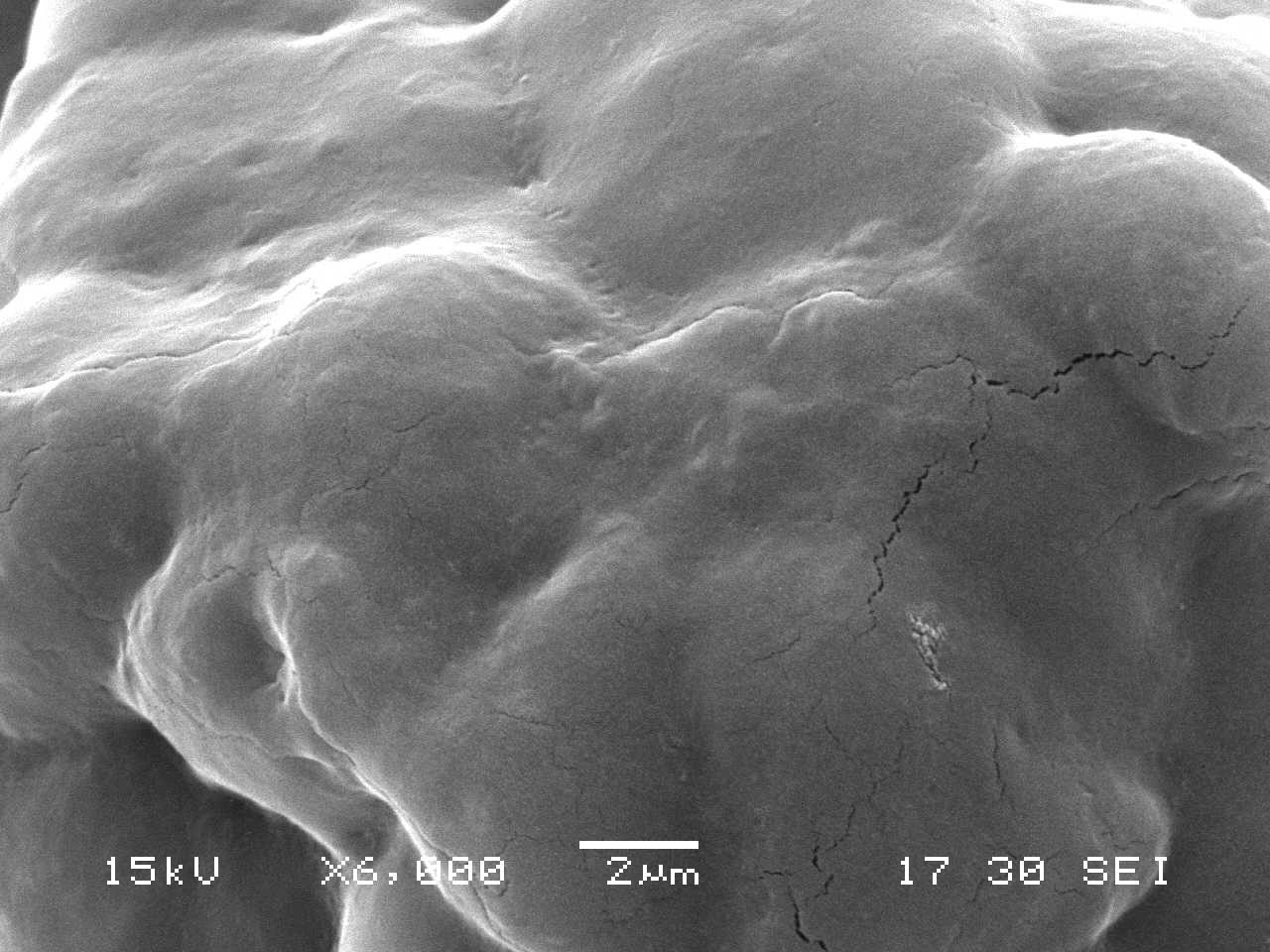

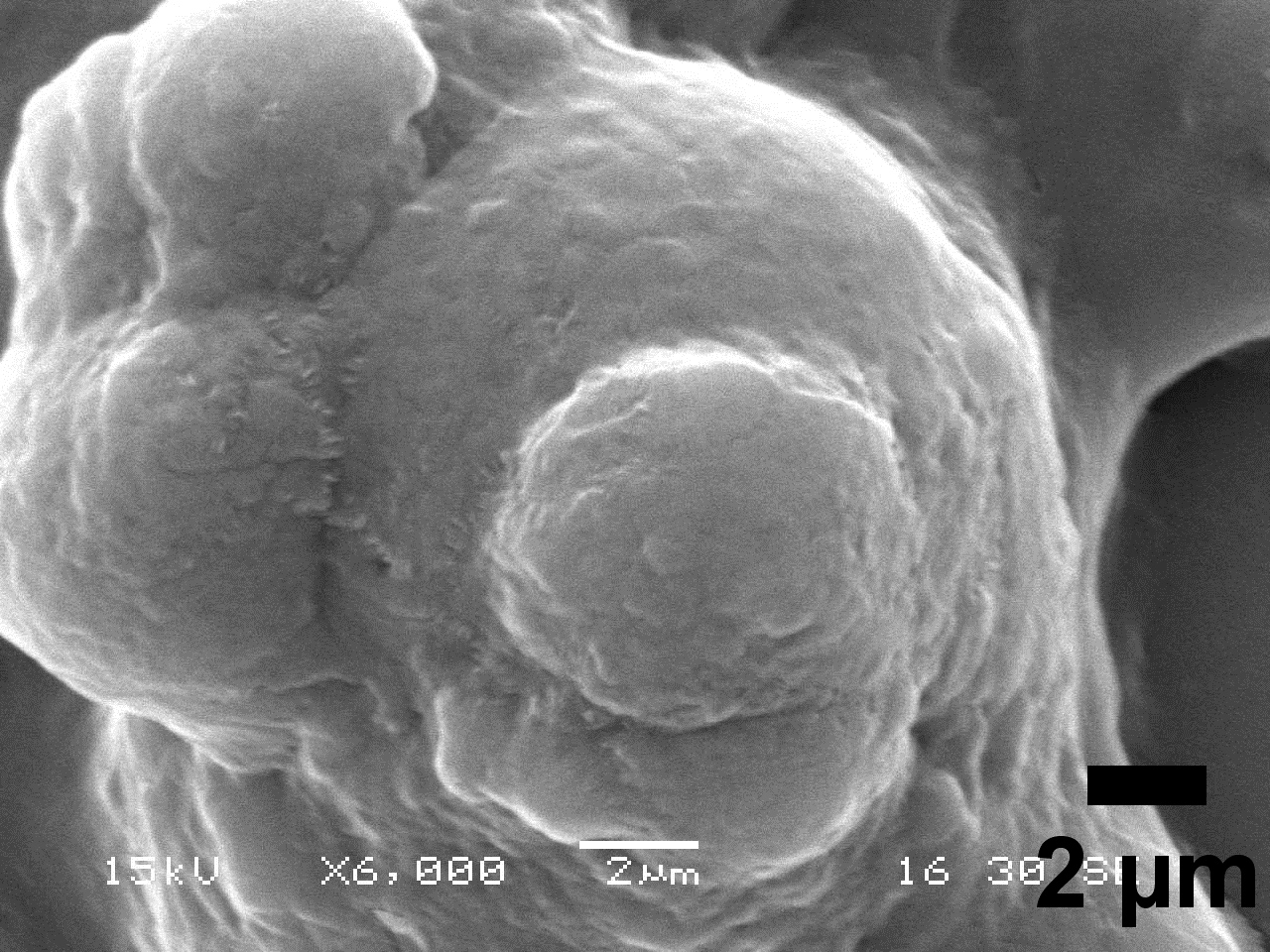


2×10^5^ cells/mL

4×10^5^ cells/mL

8×10^5^ cells/mL

4×10 ^5^ cells/mL

50 rpm

**Fig. S1.** High-magnification (6000 ×) SEM analyses of bACs aggregates after 1, 3 and 5 d of culture in spinner flask. Scale bar=2 μm.


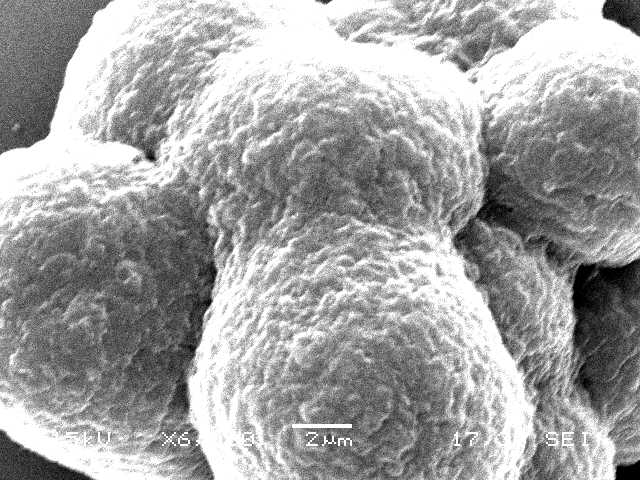

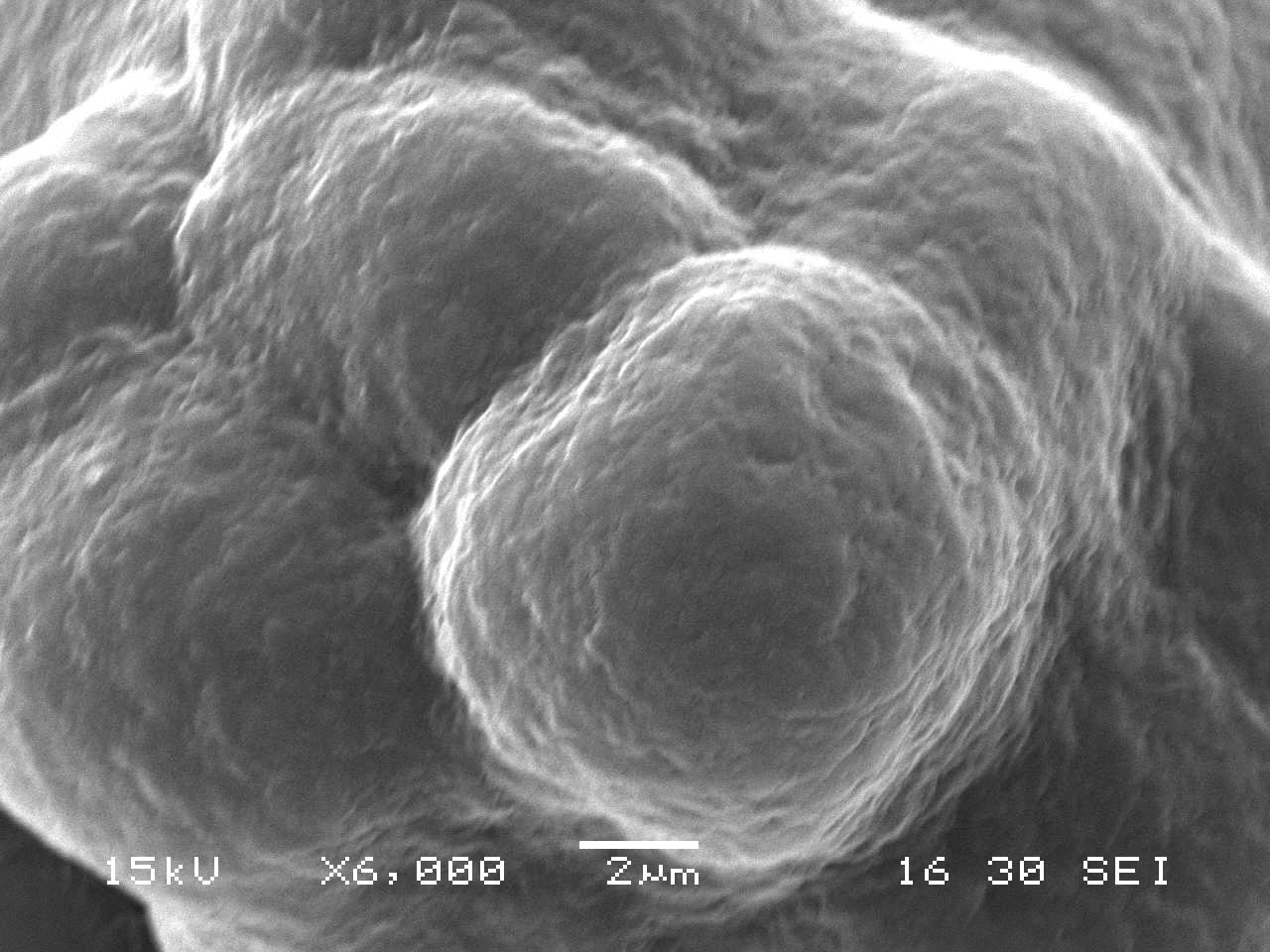

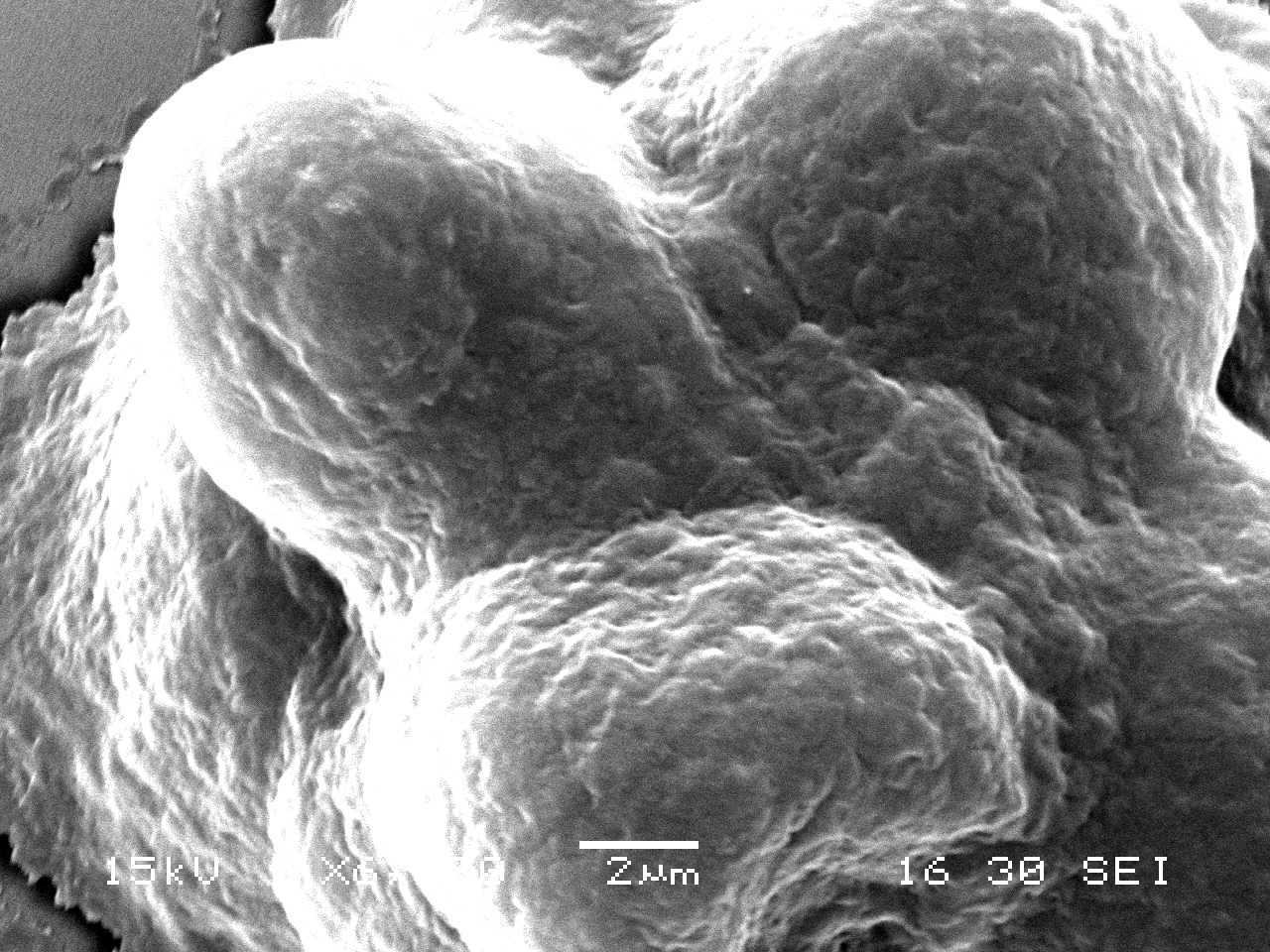

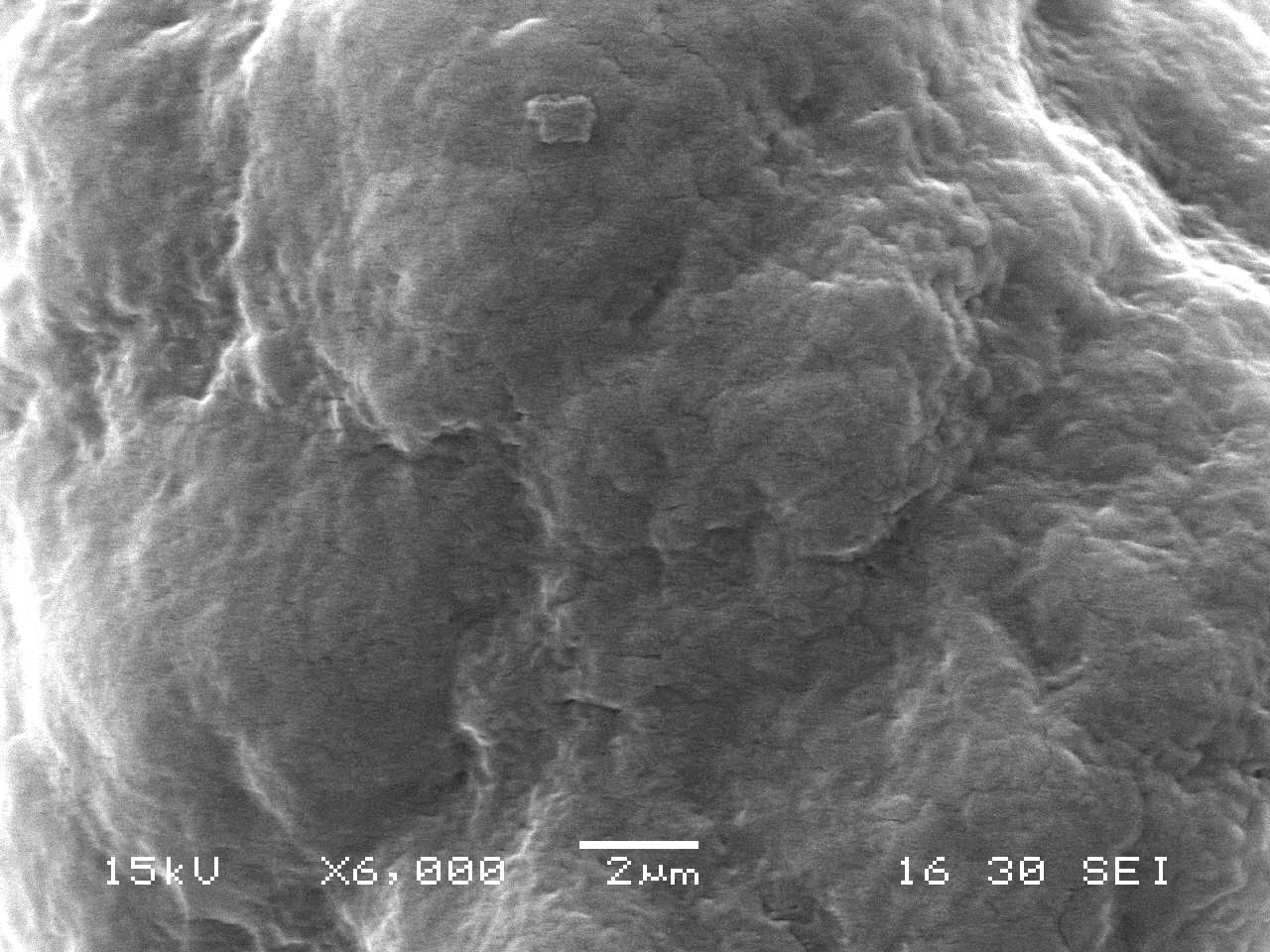

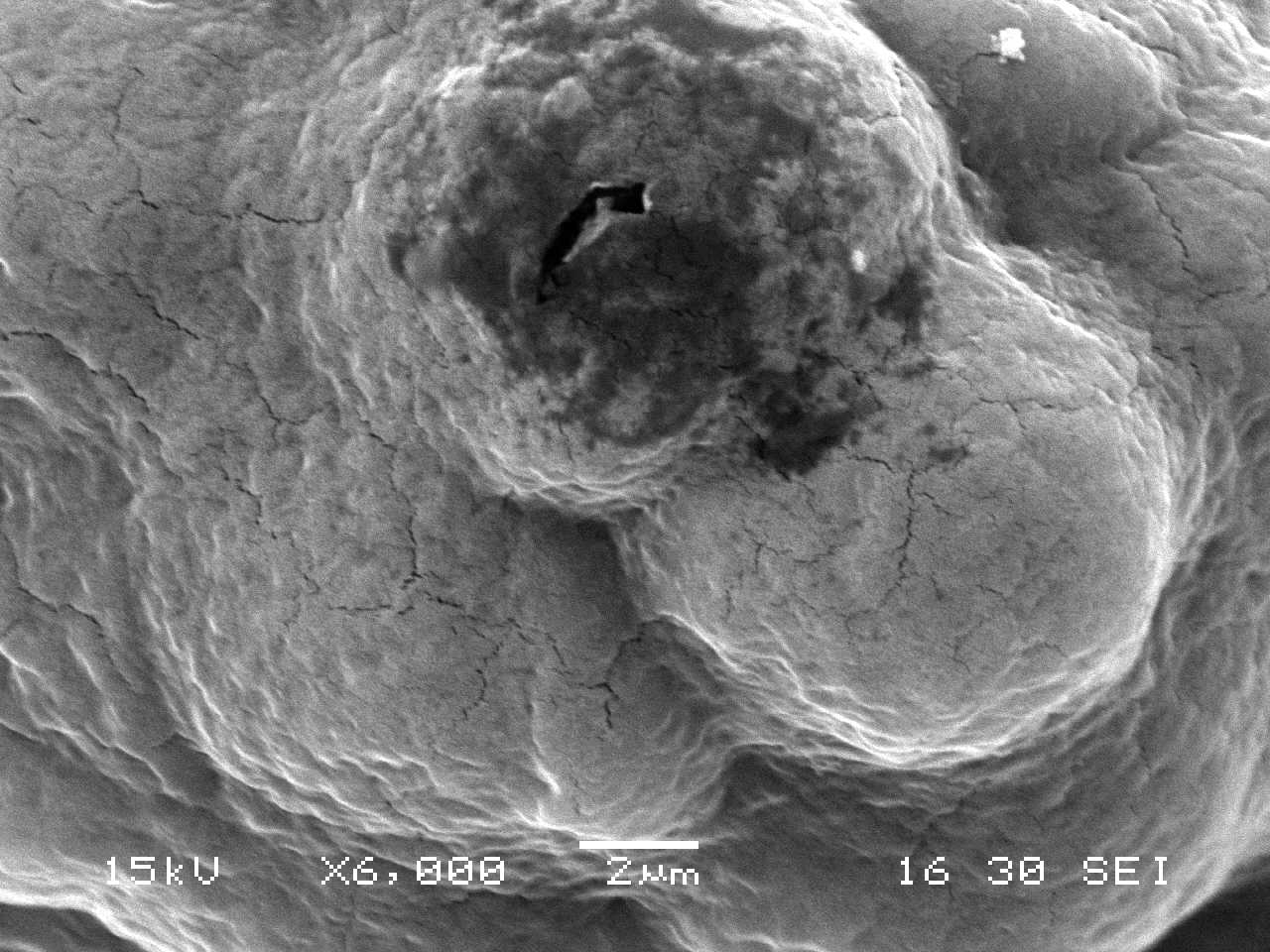

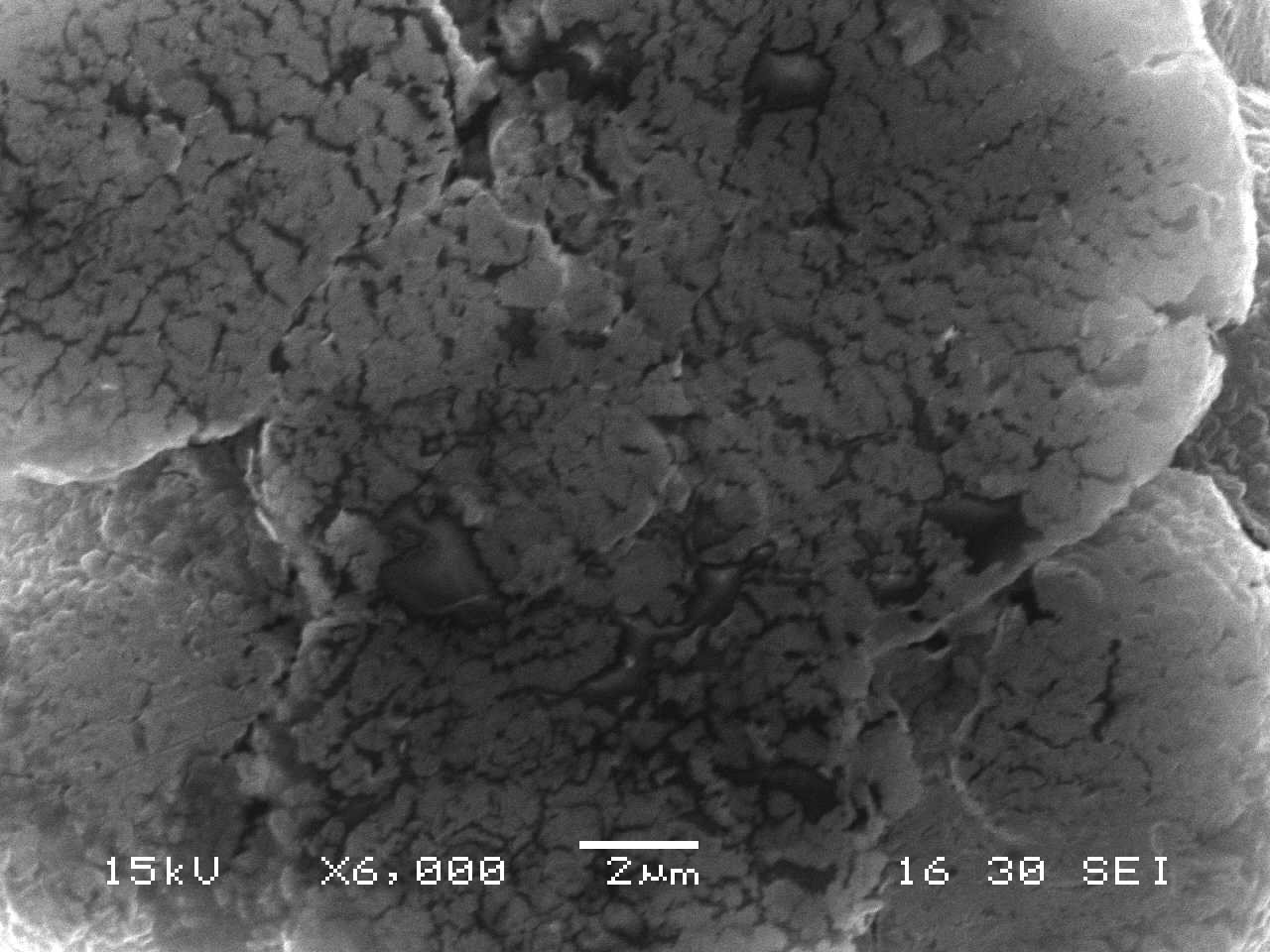

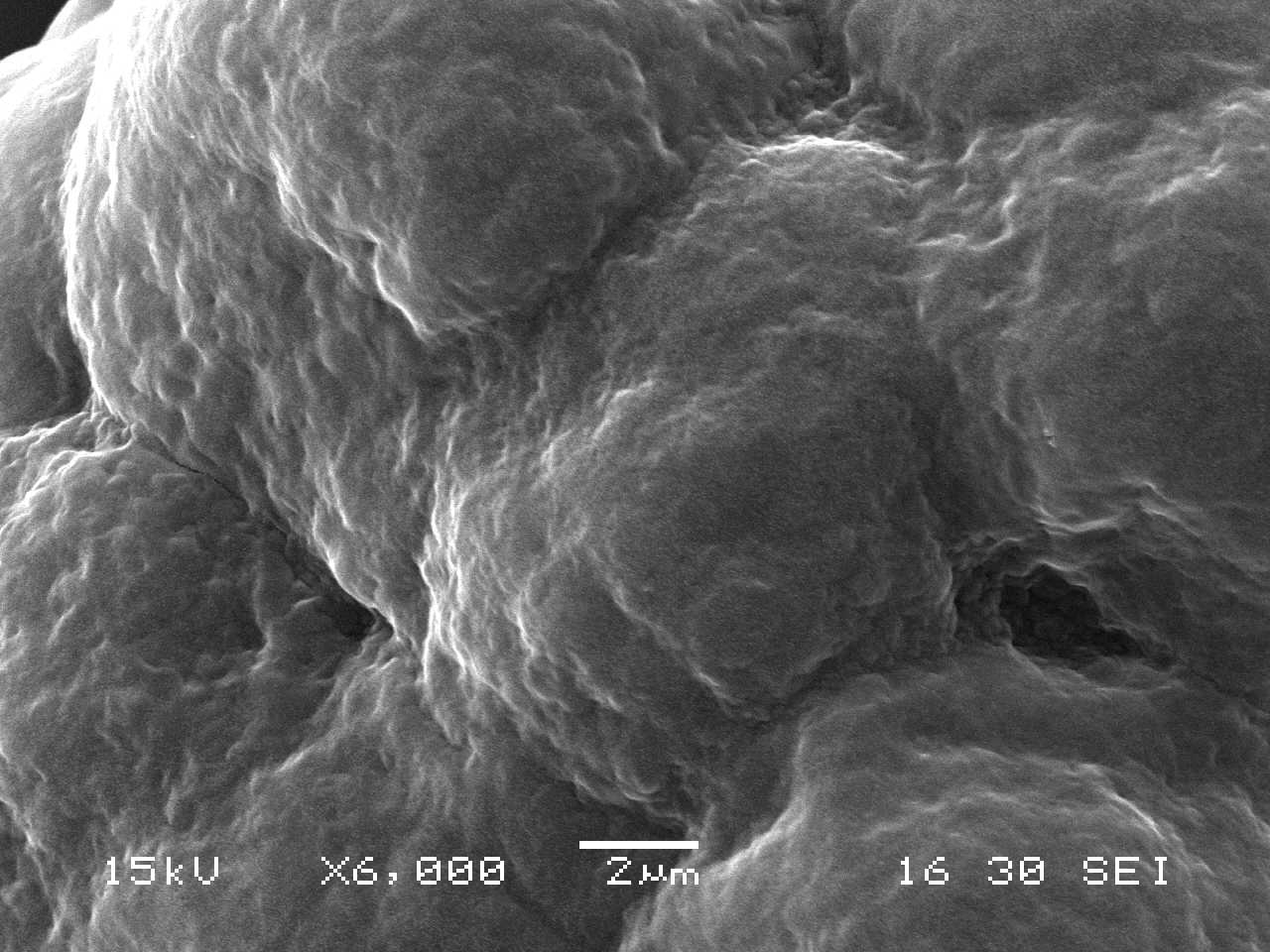

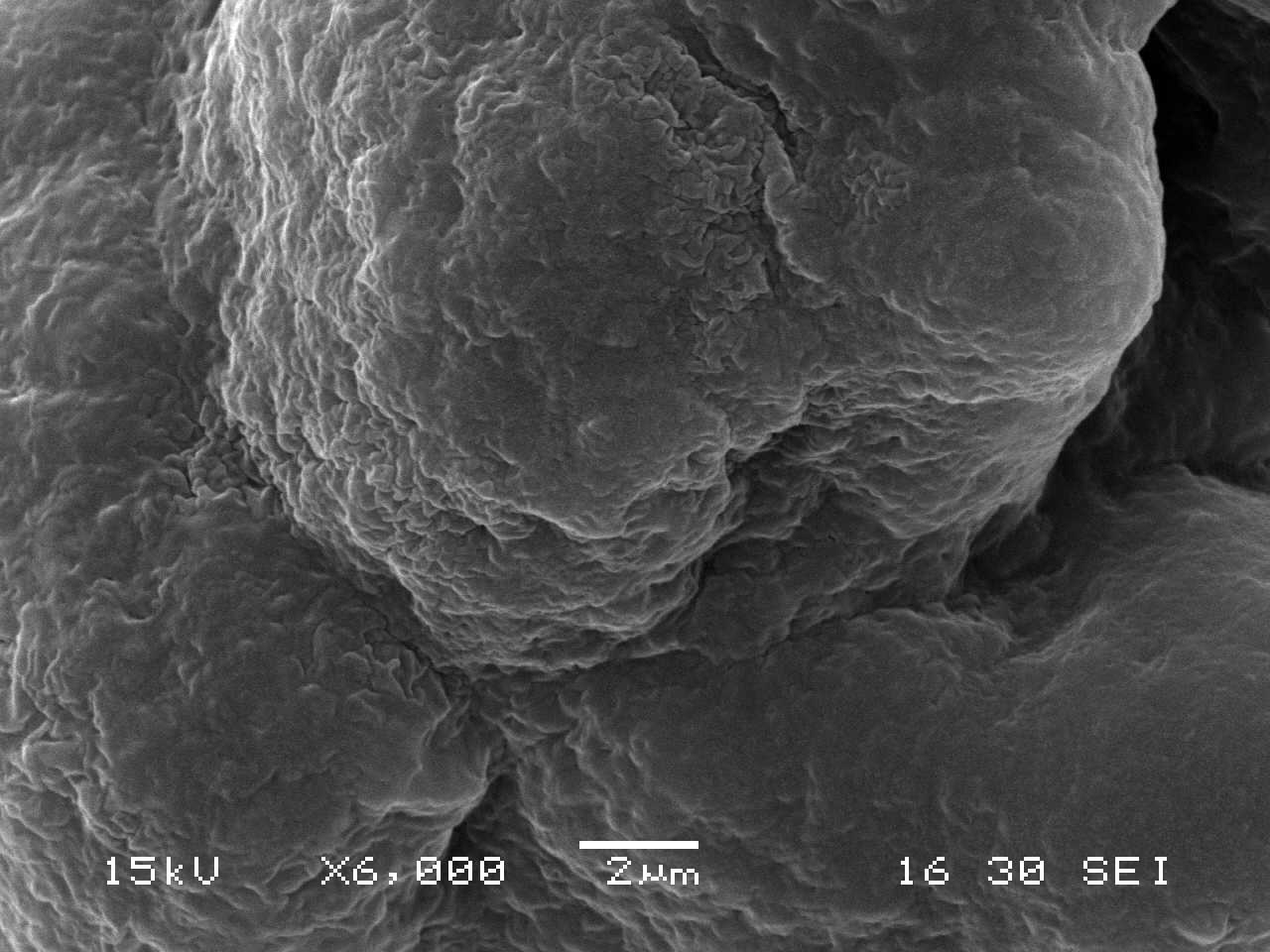

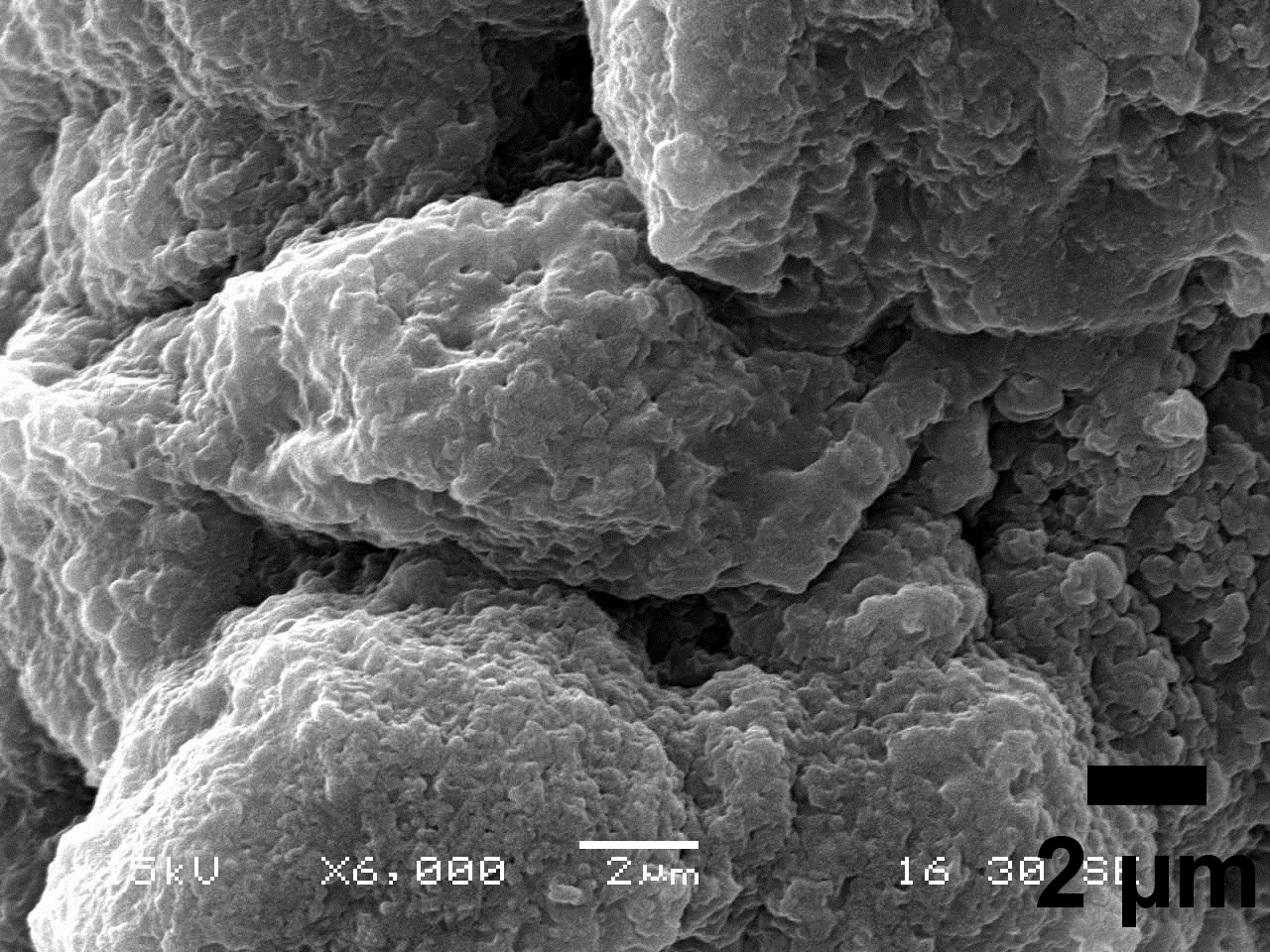


1 d

3 d

5 d

40 rpm

45 rpm

50 rpm

2×10^5^ cells/mL

4×10^5^ cells/mL


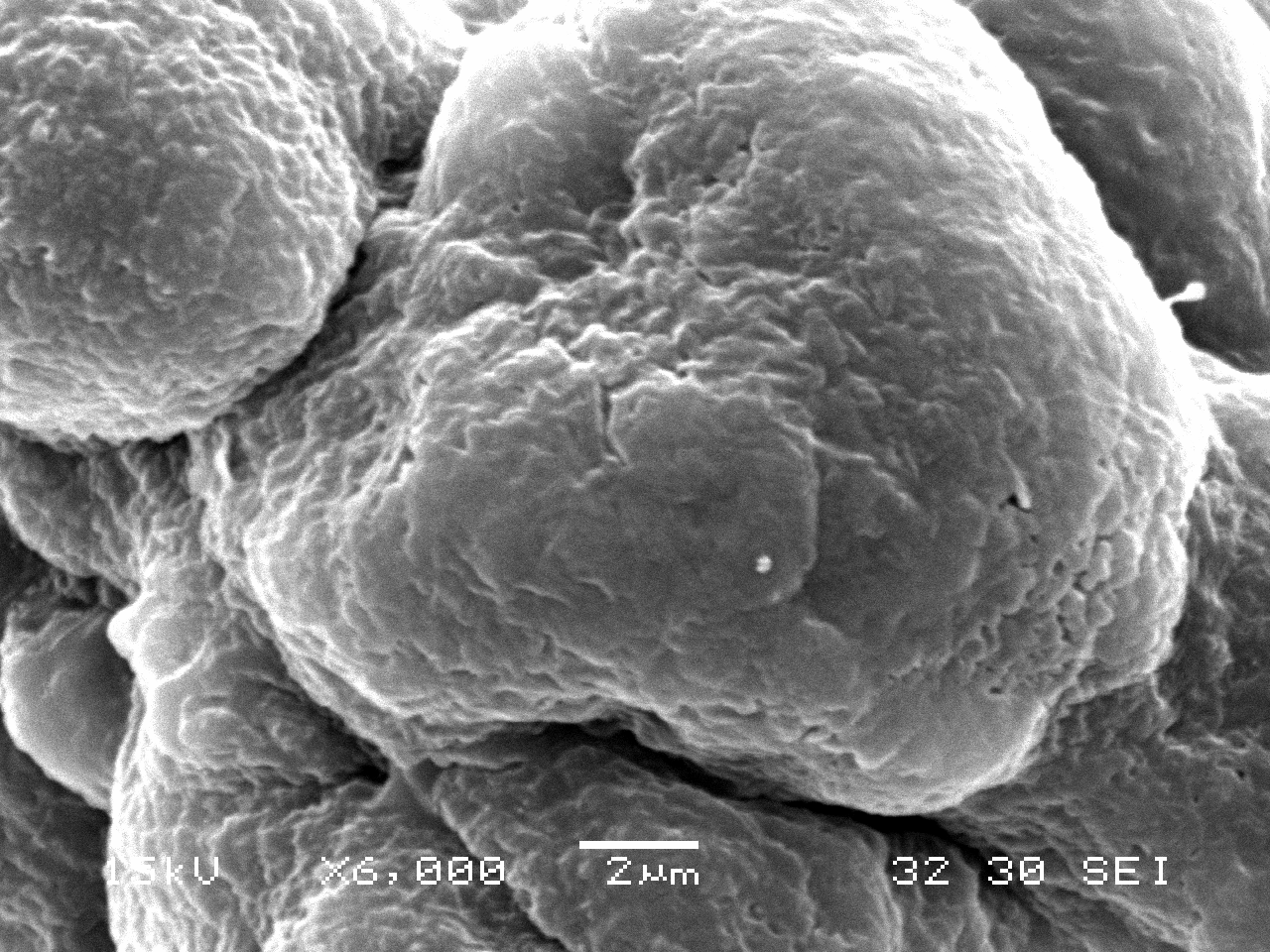

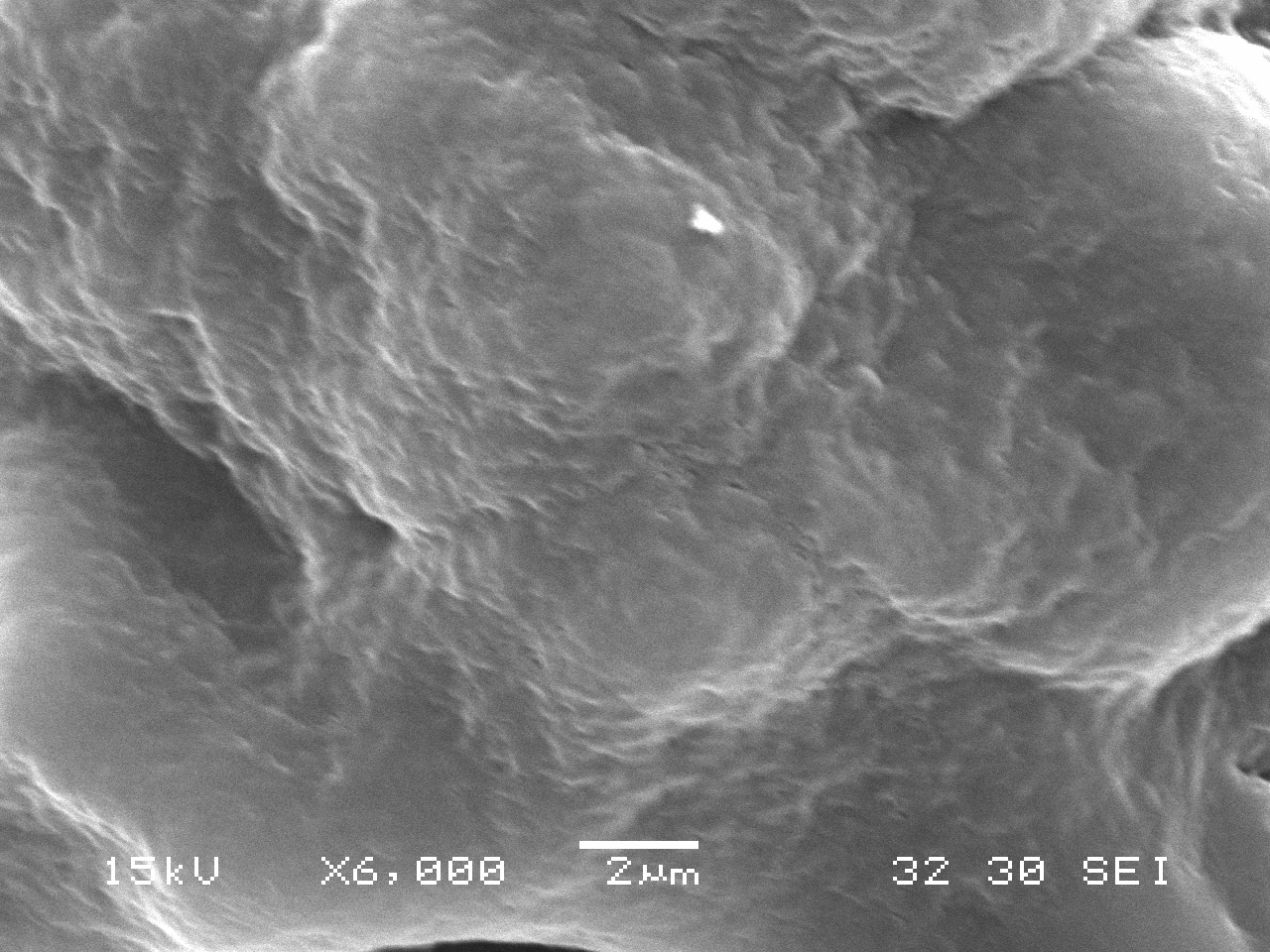

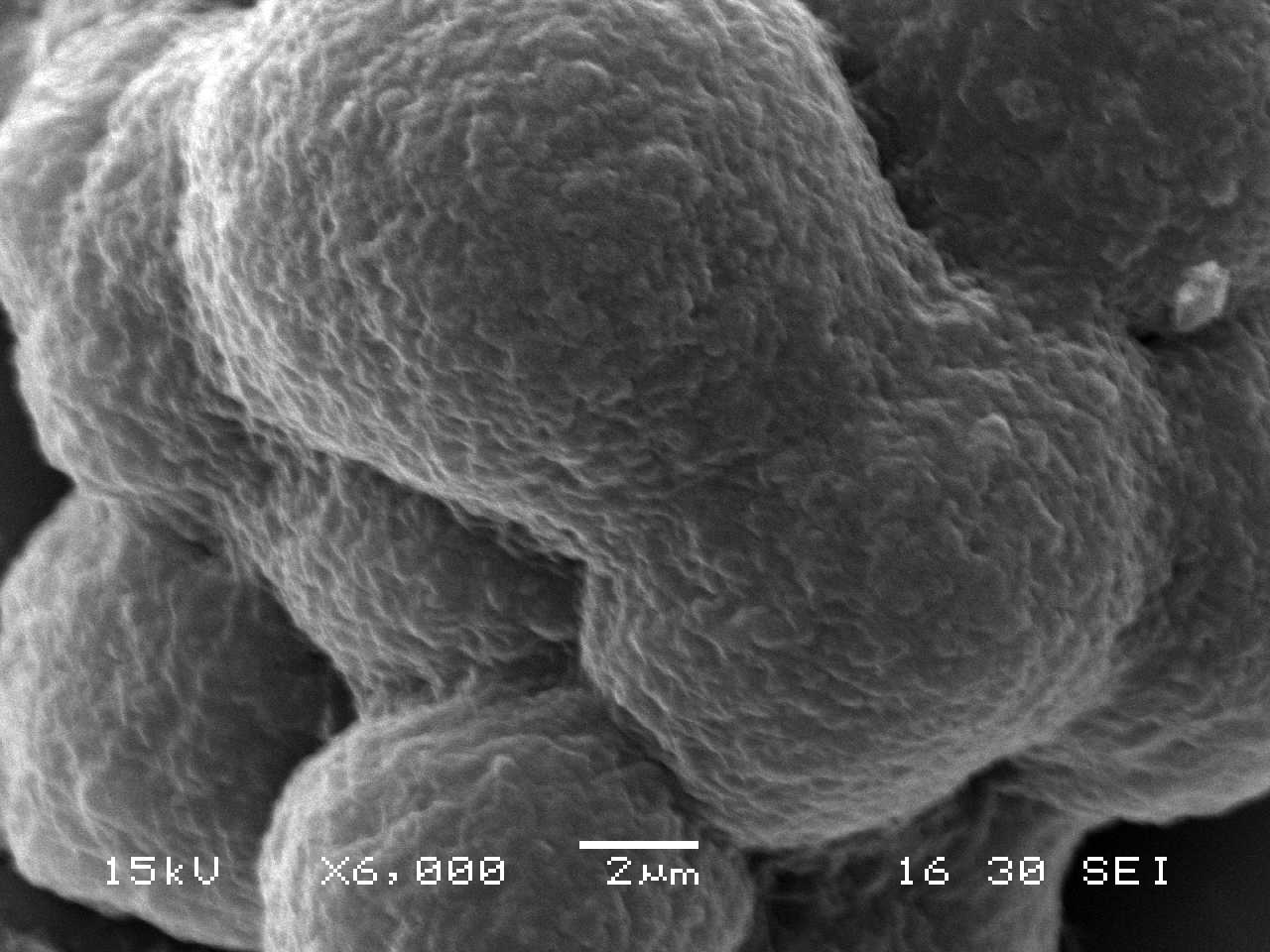

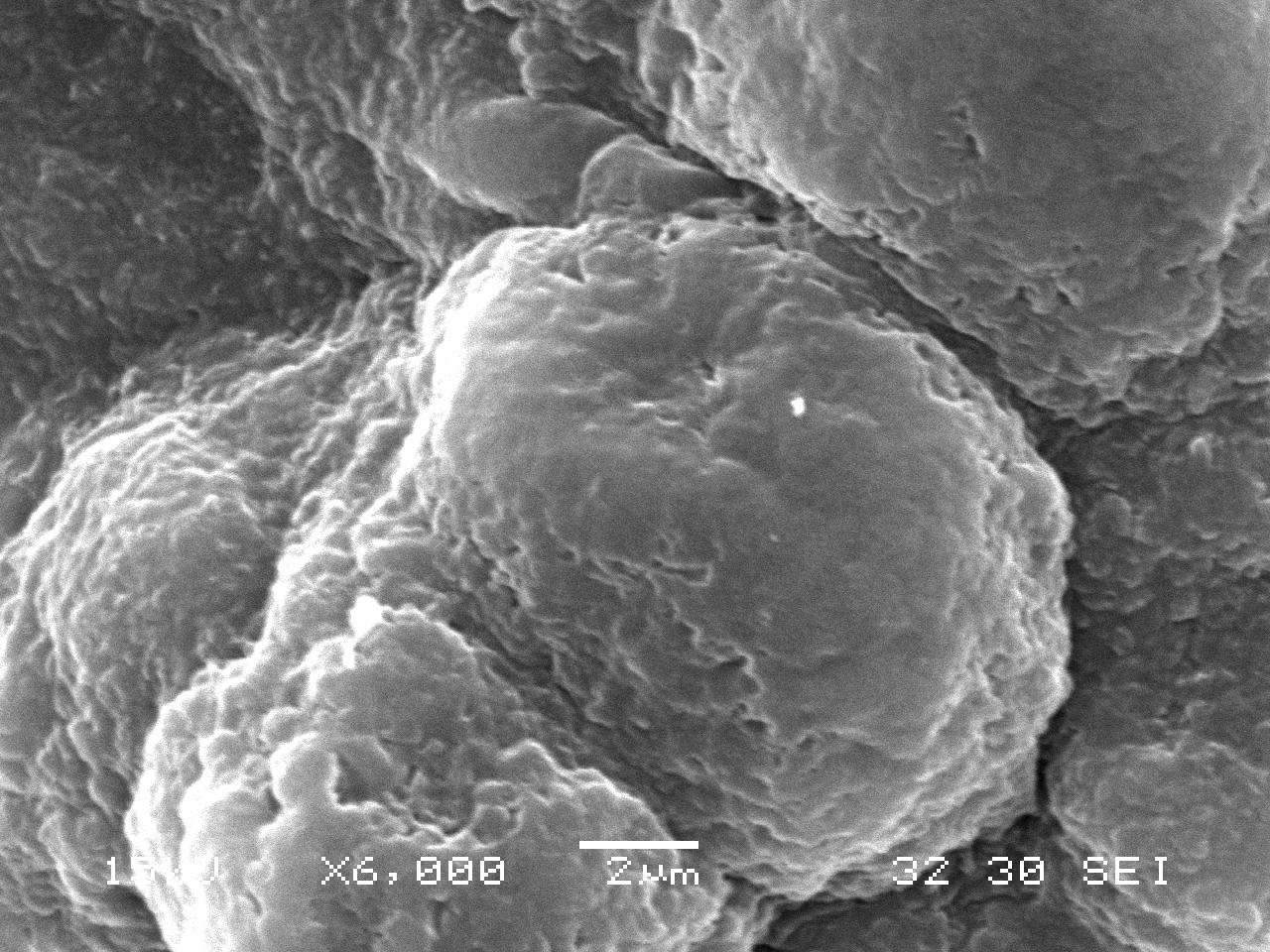

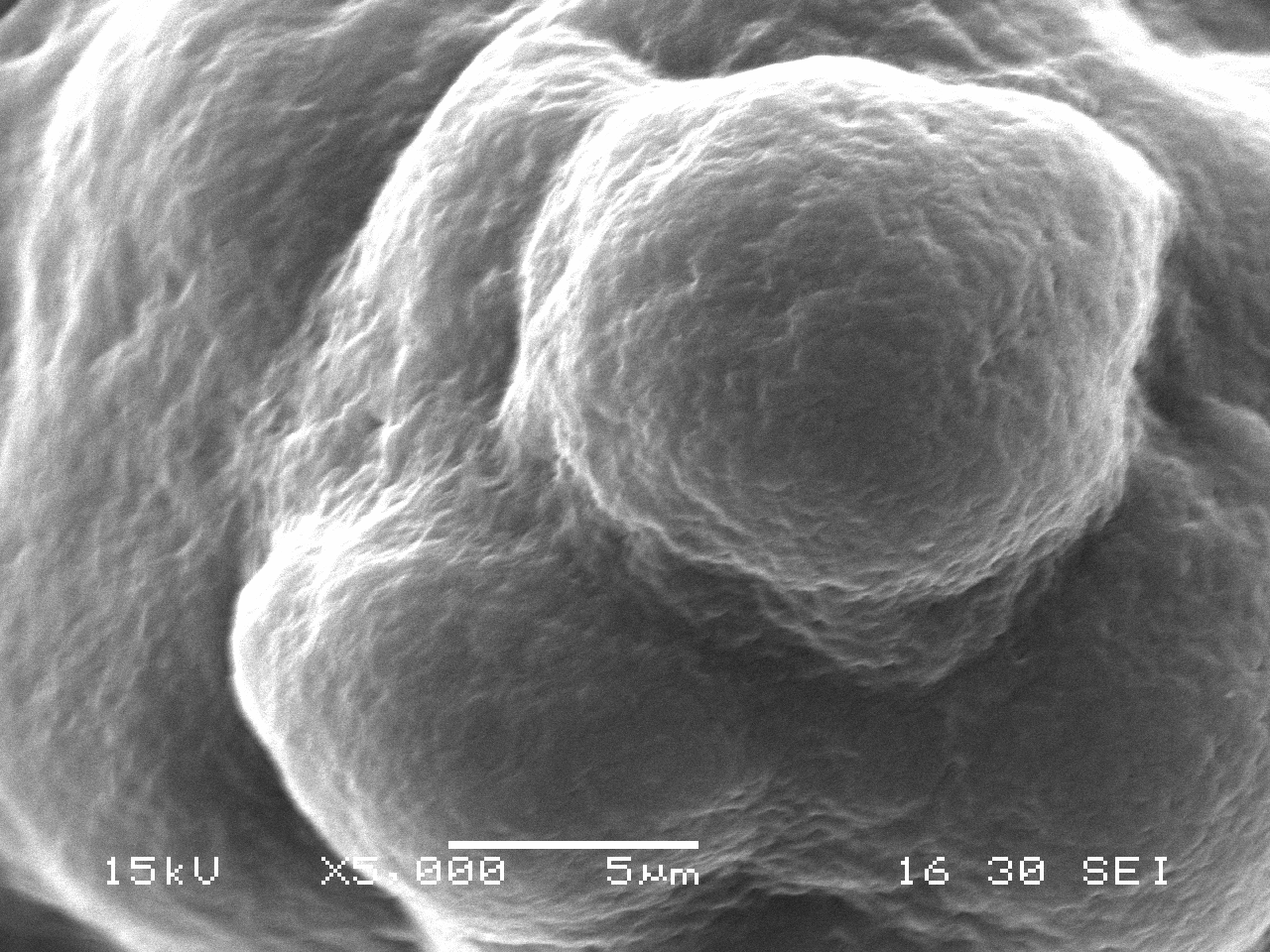

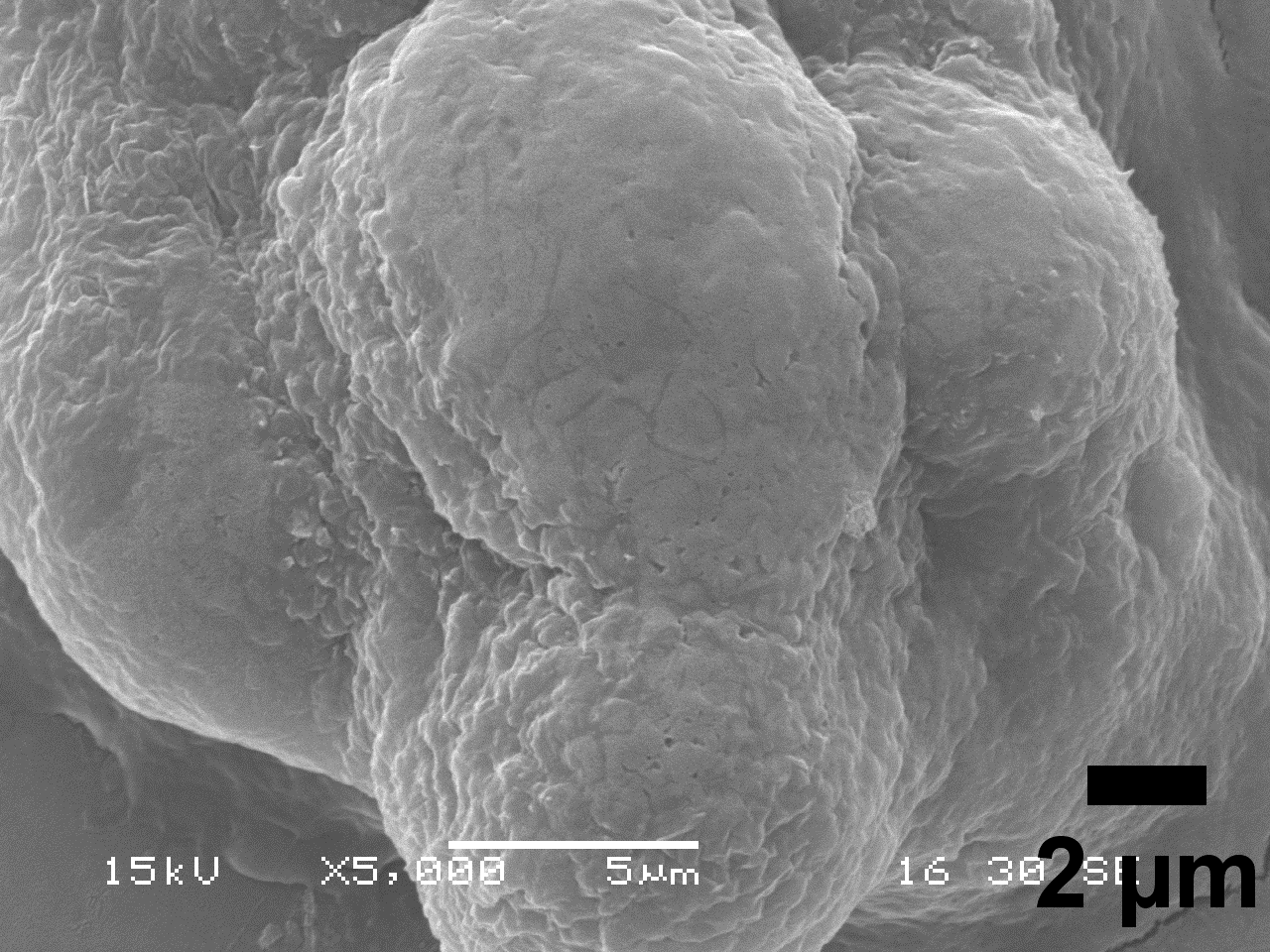


4×10 ^5^ cells/mL

45 rpm

**Fig. S2.** High-magnification (6000 ×) SEM analyses of rMSCs aggregates after 1, 3 and 5 d of culture in spinner flask. Scale bar=2 μm.

A


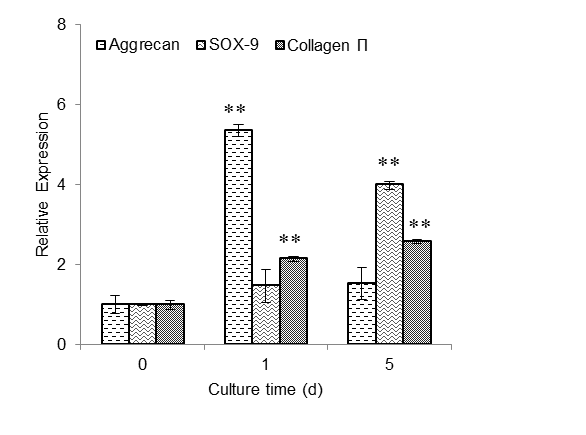

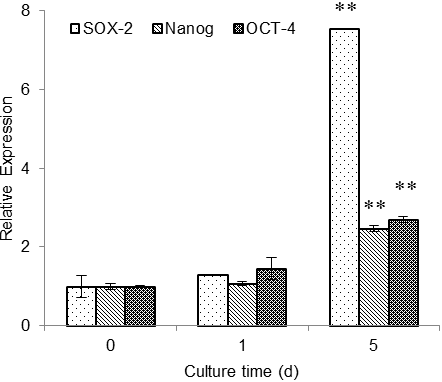


B

**Fig. S3.** Quantification of the expression levels of (A) bACs phenotype-related genes including Aggrecan, SOX-9 and Collagen II and (B) stemness-related genes of rMSCs including SOX-2, Nanog and OCT-4 cultured in spinner flask by qRT-PCR (n = 3). **P<0.01 compared to day 0.
